# Replaying germinal center evolution on a quantified affinity landscape

**DOI:** 10.1101/2025.06.02.656870

**Published:** 2025-06-05

**Authors:** William S. DeWitt, Ashni A. Vora, Tatsuya Araki, Jared G. Galloway, Tanwee Alkutkar, Juliana Bortolatto, Tiago B.R. Castro, Will Dumm, Chris Jennings-Shaffer, Tongqiu Jia, Luka Mesin, Gabriel Ozorowski, Juhee Pae, Duncan K. Ralph, Jesse D. Bloom, Armita Nourmohammad, Yun S. Song, Andrew B. Ward, Tyler N. Starr, Frederick A. Matsen, Gabriel D. Victora

**Affiliations:** 1 Department of Genome Sciences, University of Washington, Seattle, WA, USA; 2 Computational Biology Program, Fred Hutchinson Cancer Research Center, Seattle, WA, USA; 3 Laboratory of Lymphocyte Dynamics, The Rockefeller University, New York, NY, USA; 4 Present address: BioMedicine Design, Pfizer, Cambridge, MA, USA; 5 Department of Integrative Structural and Computational Biology, The Scripps Research Institute, La Jolla, CA, USA; 6 Howard Hughes Medical Institute, New York, NY, USA; 7 Howard Hughes Medical Institute, Seattle, WA, USA; 8 Department of Physics, University of Washington, Seattle, WA, USA; 9 Paul G. Allen School of Computer Science and Engineering, University of Washington, Seattle, USA; 10 Department of Applied Mathematics, University of Washington, Seattle, WA, USA; 11 Department of Statistics, University of California, Berkeley, CA, USA; 12 Computer Science Division, University of California, Berkeley, CA, USA; 13 Department of Biochemistry, University of Utah School of Medicine, Salt Lake City, UT, USA; 14 Department of Statistics, University of Washington, Seattle, WA, USA

## Abstract

Darwinian evolution of immunoglobulin genes within germinal centers (GC) underlies the progressive increase in antibody affinity following antigen exposure. Whereas the mechanics of how competition between GC B cells drives increased affinity are well established, the dynamical evolutionary features of this process remain poorly characterized. We devised an experimental evolution model in which we “replay” over one hundred instances of a clonally homogenous GC reaction and follow the selective process by assigning affinities to all cells using deep mutational scanning. Our data reveal how GCs achieve predictable evolutionary outcomes through the cumulative effects of many rounds of imperfect selection, acting on a landscape shaped heavily by somatic hypermutation (SHM) targeting biases. Using time-calibrated models, we show that apparent features of GC evolution such as permissiveness to low-affinity lineages and early plateauing of affinity are best explained by survivorship biases that distort our view of how affinity progresses over time.

## INTRODUCTION

The rapid pace of pathogen evolution presents a formidable challenge to slower-evolving hosts. In response to this pressure, vertebrates have developed equally dynamic immune countermeasures, including the capability to generate vast repertoires of immunoglobulins (Igs) by stochastic gene recombination and then improve the potency with which these bind to antigen by rapid somatic evolution^1,2^. This “maturation” of antibody affinity occurs in germinal centers (GCs), clusters of rapidly dividing B cells that arise in secondary lymphoid organs upon infection or immunization^2–4^. There, B cells perform iterative rounds of stochastic somatic hypermutation (SHM) on their *Ig* genes, each followed by selective expansion of mutant lineages with improved antigen binding. This patently Darwinian evolutionary process can generate antibodies with remarkably high affinities towards virtually any antigen. Predicting and controlling the outcome of GC evolution is therefore a major goal for vaccination efforts^5,6^.

Over the past several decades, work by a number of groups has led to a broad understanding of the cellular and molecular mechanisms that drive evolution in the GC^2–4^. *Ig* sequencing analyses show that B cell affinity increases progressively with time^7,8^, and mechanistic studies have demonstrated a role for competition among B cells for help from a limited number of Tfh cells in driving the preferential expansion of high-affinity GC members^9^. Nevertheless, the dynamical evolutionary mechanisms of GC selection remain poorly understood. For example, “clonal bursts”—sudden, drastic proliferative sprees in which the entire GC is taken over by the descendants of a single cell in a matter of a few days—occur stochastically in some GCs but not others, in a manner that is not easily predictable based on the affinity of the bursting GC B cells themselves^10^. Likewise, empirical studies have documented the coexistence of high- and very low-affinity GC B cells within the same lymph node or even the same individual GC^10–14^, suggesting that GC selection might be permissive to retaining such cells; however, such permissiveness fits poorly with our current mechanistic understanding of affinity-based selection in the GC^2^. Finally, although it is well established that higher affinity leads to improved B cell competitiveness in the GC, the relationship between affinity and fitness—i.e., how much more likely a B cell is to persist given a certain gain in affinity—has not been defined at the quantitative level.

In addition to their importance to immunity, GCs provide a unique system in which to study evolution itself. Unlike most natural evolutionary processes, GC selection is highly focused: only two genes (*Igh* and either *Igk* or *Igl*) that mutate at an exceptionally high rate^15–17^ are subject to selection essentially on a single trait—the ability to bind antigen and present it to T cells^9^. Such focus, combined with the GC’s compressed timescale, experimental tractability and replicability, and critical role in immunity, makes the system ideally suited for studying predictability in evolution^18^. Here, we present the results of such an experiment, in which we traced the phylogenetic histories of more than one hundred iterations of a clonally homogeneous GC reaction, assigning affinities to each cell based on their *Ig* sequence. We found that, although phylogenetic outcomes of GC selection were highly variable, the ability of GCs to select for high-affinity mutants was both highly sensitive and remarkably consistent. There was no evidence that clonal bursts are exclusive or even major drivers of affinity maturation. Instead, persistent selection for higher-affinity lineages in an environment of high stochasticity underlies the reproducible increase in Ig affinity at the population level. Moreover, by inferring an explicit fitness landscape that relates B cell fitness to Ig affinity, we show that both the apparent permissiveness of GCs to low-affinity lineages and the appearance of an early plateau on affinity maturation can be explained by survivorship biases that distort phylogenies of cells that survive to be sampled.

## RESULTS

### Parallel replay of evolutionary trajectories in clonally identical GCs

A quantitative analysis of GC selection requires the ability to generate replicated GC evolutionary trajectories starting from the same pool of founder B cells. To achieve this, we established a simplified system in which GCs are composed entirely of B cells carrying the same pre-rearranged *Igh* and *Igk* genes, ensuring identical starting specificity and affinity. We chose a B cell receptor (BCR) specific for the model antigen chicken IgY (clone 2.1), which we characterized extensively in previous work^10,19^. This was the dominant clone in a GC that contained a very large clonal burst (accounting for >80% of cells) 10 days after immunization with IgY^10^. We engineered mice carrying the rearranged, unmutated *Igh* and *Igk* genes of clone 2.1 in their respective loci, which we refer to as “chIgY” mice (Fig. S1A,B). We further bred these mice to a strain carrying a ubiquitously expressed photoactivatable (PA)GFP transgene to enable isolation of B cells from individual GCs within a LN by *in situ* photoactivation^9,10^. To generate fully monoclonal GCs, we adoptively transferred 5 x 10^5^ purified chIgY B cells (*Igh*^chIgY/+^.*Igk*^chIgY/+^.PAGFP-tg) into CD23-Cre.*Bcl6*^flox/flox^ recipients^20,21^, which cannot generate GCs from endogenous B cells because these lack the GC master transcription factor Bcl6.

**Figure 1.**
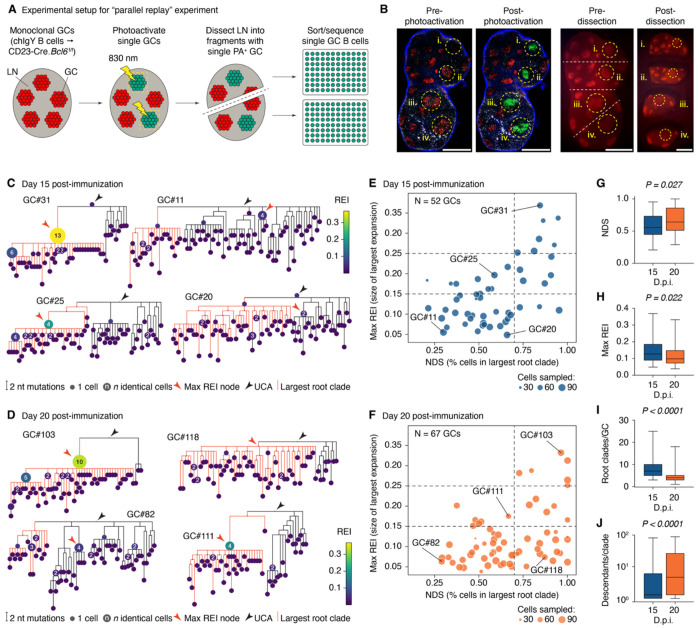
Parallel replay of germinal center evolution. (**A**) Experimental design. Monoclonal germinal centers were generated by transfer of IgY-specific B cells into GC-deficient (CD23-Cre.*Bcl6*^flox/flox^) mice, which are then immunized with IgY to generate GCs. At 20 dpi, one or more individual GCs per LN were photoactivated under a multiphoton microscope. LNs were then dissected into fragments containing a single photoactivated GC, and photoactivated GC B cells were sorted into 96-well plates for *Ig* sequencing. (**B**) Example of GC photoactivation. Left, tiled multiphoton images showing photoactivation of four individual GCs in one node. Image is one Z-plane. Right, fluorescent stereoscope images of LNs prior to and after dissection into fragments. Dotted lines and roman numerals indicate photoactivated GCs. Scale bar, 0.5 mm. (**C,D**) Examples of phylogenetic trees for GCs sequenced at 15 and 20 dpi. (**E,F**) Distribution of NDS and REI scores for GCs from 15 and 20 dpi; each symbol represents one GC scaled according to the number of B cells sequenced. (**G-J**) Comparison of phylogenetic features of GCs obtained at 15 and 20 dpi. Bar is median, boxes are 25-75%, whiskers are range. Units are GCs (H-J), and root clades (K). Data are for 52 GCs (3,758 cells; 413 root clades) from 18 mice, 2 independent experiments for 15 dpi and 67 GCs (4,986 cells; 271 root clades) from 6 mice in 2 independent experiments for 20 dpi. P-values are for Mann-Whitney U test.

We immunized recipient mice subcutaneously with IgY in alum in the footpads and base of tail to generate GCs consisting almost exclusively of donor cells within an otherwise polyclonal host (Fig. 1A and Fig. S1C). GCs from these mice were analyzed at two time points, 15 and 20 days post-immunization (dpi)—roughly 5 and 10 days after GCs peak in cell numbers at approximately 10 dpi. One day prior to analysis, we injected host mice with a Cy3-labeled antibody to CD35 to fluorescently mark the follicular dendritic cell (FDC) networks around which GCs coalesce; on the day of analysis, we explanted draining LNs, photoactivated 1-4 individual GCs per node using the labeled FDC networks as guidance, sliced nodes into segments containing a single photoactivated GC when necessary, and then sorted photoactivated B cells from each segment into 96-well plates for variable region *Igh* and *Igk* sequencing (Fig. 1B and Fig. S1C). Using this approach, we obtained paired *Ig* sequences for 8,744 GC B cells from 119 replicate GCs (15 dpi: 3,758 cells from 52 GCs (18 mice), median 75 (range 30-87) cells/GC; 20 dpi: 4,986 cells from 67 GCs (6 mice), median 78 (25-94) cells /GC). Somatic mutations were present in most cells (only 10 cells and 1 cell were unmutated at 15 and 20 dpi, respectively). Median SHM load was 5 (range, 0-18) and 7 (0-19) nucleotide mutations per cell at 15 and 20 dpi, respectively.

To examine the reproducibility of GC evolution across these 119 replicates, we first inferred phylogenetic trees for each GC using the *gctree* package^22–24^, built specifically to resolve phylogenies derived from single-GC data. This revealed a wide variety of tree topologies, ranging from phylogenies that were dominated by large clonal bursts to highly branched trees consisting of multiple competing lineages arising directly from the unmutated ancestor (Fig. 1C,D and Fig. S2). We quantified this variation using two metrics (Fig. S1E). First, we calculated the extent to which a GC was dominated by its largest lineage using the “normalized dominance score” (NDS), corresponding to the fraction of cells in a GC accounted for by its largest root clade (defined as any monophyletic group emanating directly from the unmutated ancestor, represented in red in Figs. 1C,D). This metric is meant to be loosely equivalent to the color-based NDS used in our previous quantification of GC clonality using “Brainbow” alleles^10^. Second, we computed a metric for clonal burst size we call the “recent expansion index” (REI), which quantifies how much of a phylogeny is contributed by a given node and its recent descendants (Fig. S1E). As clonal bursting involves a transient cessation of SHM^25,26^, the REI is a weighted count of descendants with a decay factor *τ* that exponentially downweights descendants as mutational distance from the focal node increases. Thus, high REI scores are indicative of nodes that underwent mutation-free expansion more recently. For each GC, we plot the value for the highest-REI node in the phylogeny (max REI).

GCs displayed a wide range of lineage dominances and burst sizes at both time points (Fig. 1E,F). Those with low NDS and max REI had multiple, evenly competitive root clades (e.g., GCs #11, #82), indicating failure of any single clade to establish dominance. At the other extreme were large clonal bursts (e.g., GCs #31, #103), the strongest of which were able to eliminate most competing clades. Intermediate cases included lineages that expanded in a burst-like manner but not to a sufficient extent to eliminate their competitors (e.g., GCs, #25, #111). Although the frequency of clonal bursts (top-right sector, e.g., GC #31) was similar between the two time points (7/52 at 15 dpi vs. 6/67 at 20 dpi), the fraction of GCs with high NDS but low max REI (bottom-right sector; e.g. GC #118) increased significantly at 20 dpi (3/52 vs. 18/67; p_Fisher_ = 0.0031). This was accompanied by slight increases in NDS and in the number of root clades per GC, accompanied by slight decreases in max REI and the number of cells per root clade, from 15 to 20 dpi (Fig. 1G–J). This shift aligns with expected GC evolutionary dynamics: as established clonal bursts “age,” their descendants accumulate mutations, reducing max REI. On the other hand, root-clade diversity (equivalent to the diversity of V(D)J rearrangements in a polyclonal setting) is not restored, such that NDS remains high.

We conclude that evolutionary trajectories of GCs are highly variable even when founder populations are identical at the *Ig* sequence level. Whereas at their strongest, clonal burst-type events were powerful enough to eliminate virtually all competing lineages in a GC, such events were relatively rare, and most GCs were capable of sustaining several large lineages in parallel. Kinetic analysis showed progression towards fewer and larger lineages, detectable even over a window of five days. Thus, a simplified monoclonal model recapitulates the range of outcomes observed for polyclonal GCs^10^.

### Determining the effects of somatic mutation on antigen binding

**Figure 2.**
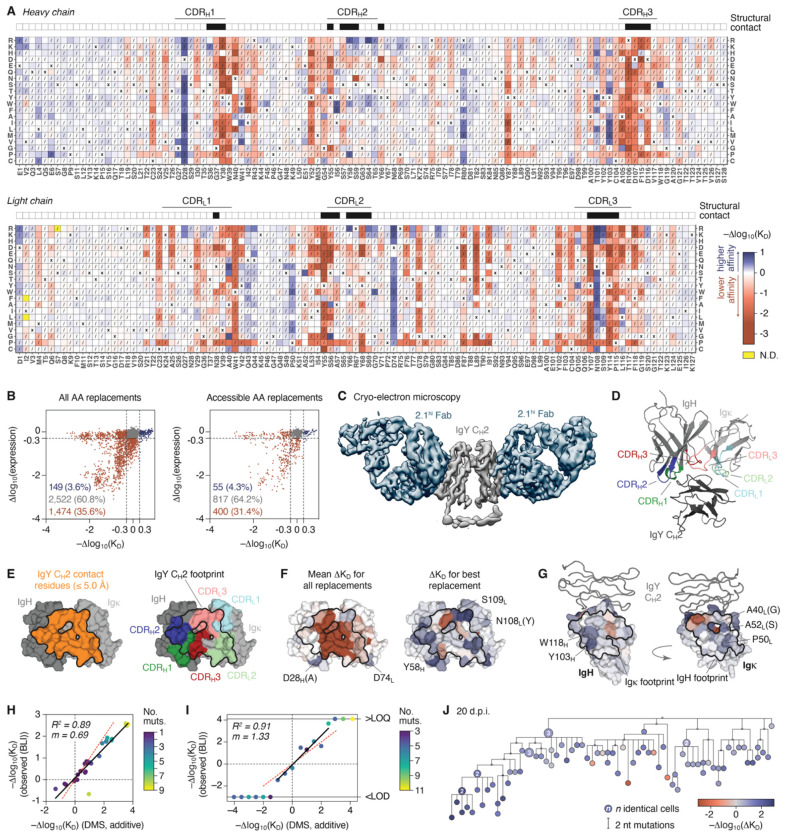
Deep mutational scanning and structure of clone 2.1. (**A**) Heatmaps showing the effects of individual amino-acid replacements on binding of 2.1 scFv to chicken IgY by yeast display. Each square represents a different replacement. Squares marked with “X” indicate the original amino acid in clone 2.1. Squares with slashes indicate amino acid replacements distant more than one nucleotide mutation from the naive sequence. Yellow squares were not detected in the DMS experiments. Antigen-antibody contact residues (intermolecular distance ≤ 5.0 Å as determined by cryo-EM) are shown as black boxes in the upper bar. See Fig. S2C for the equivalent heatmap for scFv expression. An interactive version of this heatmap is available at https://matsengrp.github.io/gcreplay/interactive-figures/mutation-heatmaps/naive_reversions_first.html; replacements can be visualized on the Fab structure at https://matsen.group/gcreplay-viz/. Data are the mean of two independent experiments. (**B**) Effects of all (left) and accessible (requiring a single nucleotide mutation) amino acid replacements on 2.1 affinity and surface expression. The number and fraction of amino acid replacements in each category (impairment, red; neutral, gray; improvement, blue) are indicated. (**C**) 4.0 Å cryo-EM reconstruction of the 2.1^hi^ Fab (D28_H_A, K49_H_R, S64_H_G, A40_L_G, Y42_L_F, A52_L_S, Q105_L_H, N108_L_Y) complexed to IgY following local refinement using a mask around the IgY C_H_2 and 2.1^hi^ Fab regions. (**D**) Cryo-EM-derived structure of the interaction of the 2.1^hi^ V-domain with IgY C_H_2 with CDRs indicated. (**E**) Structure of the 2.1^hi^ V-domain showing the footprint (black outline) of C_H_2, defined as residues with intermolecular distance ≥ 5.0 Å. The antibody-antigen interface spans 851 Å2 (341 Å2 for V_H_ and 510 Å2 for V_κ_). (**F**) As in (E), colored by mean Δaffinity (−Δlog_10_(K_D_) for all replacements (left), or maximum Δaffinity for the best possible replacement at each position (right)). Color scale as in (A). (**G**) Structure of the 2.1^hi^ V-domain heavy (left) and light (right) chains colored by maximum Δaffinity. The approximate footprint of the opposite antibody chain (residues with ≥ 5 Å^2^ buried surface area contribution) is indicated by a black outline. IgY C_H_2 is shown as a gray ribbon. Color scale as in (A). (**H-I**) Comparison of Δaffinities predicted using the additive DMS model and BLI measurements of Fabs produced recombinantly using the same sequences. Solid black line is the linear trend; dotted red line is *x = y*. (H) 2.1 variants found frequently in prior studies of clone 2.1; (I) Affinity ladder spanning 8 orders of magnitude. LOD, limit of detection, below which BLI curve fitting was unreliable; LOQ, limit of quantitation, when Fab off-rate is too long to be determined. R^2^ and slope (*m*) calculations exclude Fabs <LOD and >LOQ. (**J**) Example GC phylogeny from the 20 dpi replay dataset (see Fig. 1), colored based on the additive DMS model.

To understand how features of clone 2.1 phylogenies related to changes in its affinity for IgY, we used deep mutational scanning (DMS) to measure the impact of virtually all possible V(D)J single-amino acid replacements to the naïve 2.1 sequence on antigen binding and Ig surface expression (Fig. S3A). We constructed a comprehensive site-saturation mutagenesis library containing 4,158 out of 4,161 possible single-amino acid replacements available to clone 2.1 (i.e., 19 alternatives for each of the 219 amino acids of the combined IgH and Igκ variable regions), which we cloned into a single-chain fragment variable (scFv) construct for yeast-surface display^27^. We then used fluorescence-activated cell sorting coupled to deep sequencing^28^ to measure the impact of each replacement on IgY-binding affinity (Δaffinity, defined as −Δlog_10_(K_D_)) and surface scFv expression levels (Δexpression, a proxy for folding stability^29,30^) (Fig. 2A and S3A–C).

As expected given the relatively tight binding of the unmutated clone 2.1 to IgY (~40 nM^10^), amino acid changes, particularly those falling within complementarity-determining regions (CDRs), were much more likely to reduce affinity for antigen than to improve it (Fig. 2A,B). This effect was strongest in the short (6-amino acid) CDR_H_3, where almost all amino acid replacements led to decreased antigen binding, and less so for the longer CDR_L_3, where multiple replacements at two positions (N108_L_ and S109_L_) led to affinity gains. DMS thus revealed the extent to which clone 2.1, and likely antibodies in general, must navigate a landscape fraught with mutations that lead to loss of either binding affinity or cell surface expression. Whereas the best available replacement improved affinity by less than one log_10_ (N108_L_R, Δaffinity = 0.91), the worst lowered affinity by 3 log_10_ (Y38_H_E, Δaffinity = −3.0; Fig. 2A). More importantly, of the 4,145 amino acid replacements assayed for both Δaffinity and Δexpression in the DMS, 1,474 (35.6%) led to at least a 0.3 log_10_ decrease in either binding affinity or scFv surface expression, while only 149 (3.6%) led to a gain in affinity of 0.3 log_10_ or greater (Fig. 2B). Similar results were obtained when only accessible replacements (those resulting from a single nucleotide mutation, non-hatched squares in Fig. 2A) were considered (400 (31.4%) deleterious and 55 (4.3%) enhancing replacements of 1,272 assayed; Fig. 2B). Thus, for every enhancing replacement clone 2.1 can make, it must avoid making roughly 10 deleterious ones. No amino acid replacements were found that led to a substantial gain in surface expression, suggesting that the stability of this antibody is close to optimal (Fig. 2B and S3C).

To understand the structural basis for our DMS results, we determined negative-stain and cryo-EM structures of an affinity-matured version of the 2.1 Fab (measured K_D_ = 6.2e–11 M) bound to IgY. In agreement with antigen truncation experiments, clone 2.1 bound to the hinge-like C_H_2 domain of the four-domain IgY constant region (Fig. S3D,E). Cryo-electron microscopy revealed that the paratope consisted of a concave pocket that contacted the outer face of IgY C_H_2 (Fig. 2C–E and Fig. S3F). Mapping the DMS data to the structure showed that residues in the central groove of the paratope, which form the bulk of the interaction surface with IgY, were highly constrained (i.e., replacements at these positions had strongly negative mean effects on Δaffinity; Fig. 2E,F and Fig. S3G). Affinity-enhancing replacements were found primarily at the periphery of the paratope (Fig. 2F) but also along the heavy-light chain interface (Fig. 2G). Replacements that substantially decreased scFv surface expression occurred in their expected positions (e.g., cysteines involved in disulfide bond formation and inward-facing hydrophobic residues and salt bridges^31^; Fig. S3H).

To estimate the affinities of GC B cells containing multiple somatic mutations, we added the –affinities associated with each of the individual amino acid replacements in its IgH and Igκ sequences. We reasoned that, since most affinity-enhancing mutations in clone 2.1 were spread out along the edges of an otherwise optimal central groove (Fig. 2F), epistatic interactions between replacements leading to non-additive effects would be limited. To validate this approach, we produced a series of recombinant monoclonal Fabs carrying selected affinity-enhancing replacements that appear frequently in clone 2.1, either alone or in combination (references ^10,19^ and our unpublished observations) and measured their binding to IgY by biolayer interferometry (BLI Supplemental Spreadsheet 1). This showed good agreement (R^2^ = 0.89, slope = 0.69) between predicted and observed values within this series (Fig. 2H). We expanded this approach to a wider range of affinities by producing a 17-step ladder comprising Fabs derived from observed chIgY B cell sequences spanning 8 orders of magnitude of affinity (from −log_10_(KD) ≅ −4.0 to +4.0). All Fabs with estimated Δaffinities below −1.0 bound too weakly to antigen to be characterized by BLI. Above this threshold, DMS-predicted and BLI-measured affinities increased linearly up to Δaffinity ≅ 2.5 (R^2^ = 0.91, slope = 1.33; Fig. 2I), after which off-rates were too long to accurately measure. Overall, the mean absolute difference in −log_10_(K_D_) between BLI measurements and DMS predictions was 0.17 for antibodies with a single mutation and 0.64 for antibodies with more than one mutation. As an estimate of the accuracy of BLI, the mean absolute difference between 9 independent BLI measurements of the unmutated Fab and the mean of these measurements was 0.20. The predicted mean Δaffinity for all antibodies tested was 1.06, compared to a measured mean of 0.88, a difference of 0.18. We conclude that adding the DMS-determined effects of individual mutations offers a reliable framework for predicting how multiple mutations affect the affinity of clone 2.1, particularly when these affinities are averaged across many cells. Figure 2J shows an example of a 20 d.p.i. GC phylogeny, colored using this model (for all trees, see Fig. S2).

### Selection of individual amino acid replacements across germinal centers

**Figure 3.**
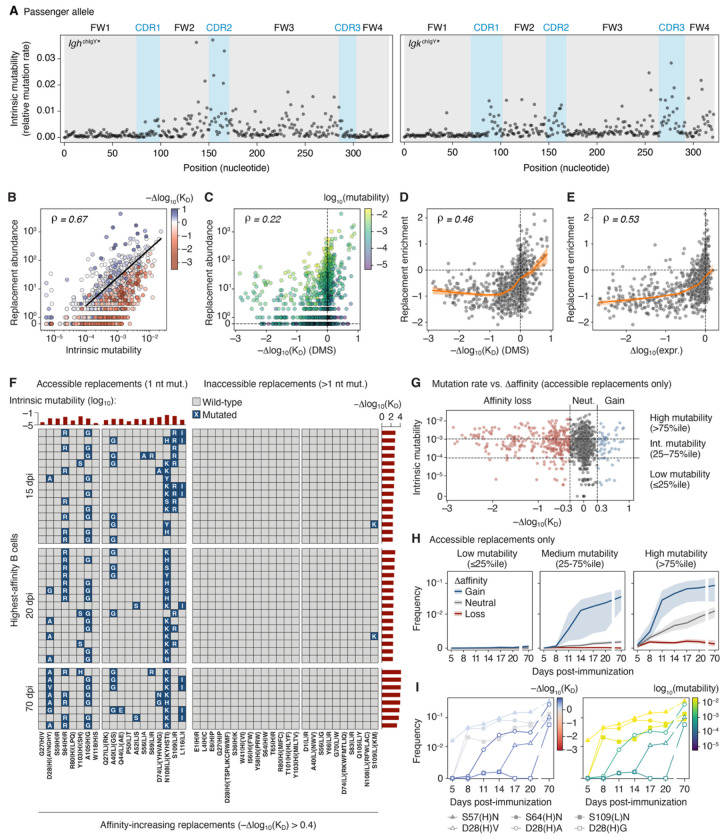
Both mutability and affinity drive accumulation of individual replacements across germinal centers. (**A**) Relative mutation rate measured for each base pair of the passenger *Igh*^chIgY^* and *Igk*^chIgY^* alleles. Each symbol represents one nucleotide position, the sum of the relative rate of mutation for each of the three non-native nucleotides is given. Each symbol represents one nucleotide position, the sum of the relative rate of mutation for each of the three non-native nucleotides is given. Data are pooled from 3 mice for *Igh* and 2 mice for *Igk*. (**B**) Correlation between relative mutation rate, calculated for each of 1,275 codon-accessible amino acid replacements based on the data in (A), and the prevalence of each of these replacements in the replay dataset. Each symbol represents one replacement, colored by the Δaffinity determined for that replacement by DMS. Black line is a Poisson regression with no link function. (**C**) Correlation between Δaffinity and the prevalence of each of these replacements in the replay dataset. Each symbol represents one replacement, colored by relative mutation rate. (**D,E**) Correlation between Δaffinity (D) and Δexpression (E) and replacement enrichment (defined as the log_10_ of the number of observed events over the number of predicted events according to a Poisson regression shown here as a line). The orange line shows a LOWESS regression with 95% confidence intervals. ρ values are for Spearman correlation. (**F**) Accumulation of affinity-enhancing replacements among the highest-affinity B cells at various time points post-immunization. Each row represents the Ig sequences of one cell (the highest-affinity B cell in its host GC). Each column represents the set (curly brackets) of affinity-enhancing replacements (affinity > 0.4 according to the DMS) available at each position, grouped into those accessible by a single nucleotide mutation (left) and those that are not (right). Grey squares indicate that a B cell has the WT amino acid at that position; blue squares indicate replacements, and the identity of the replacement made by the B cell is given in white font. Bars above the graph indicate the summed intrinsic mutability for the listed replacements calculated from the passenger allele. Bars to the right indicate the DMS-estimated Δaffinity of each B cell. (**G**) Classification of accessible amino acid replacements into categories of Δaffinity (DMS) and relative mutation rate (passenger allele), defined by the dashed lines. (**H**) Accumulation over time of replacements from the 9 categories in (F) among GC B cells sequenced at various time points after immunization as detailed in Figure S4G. Each line represents the average normalized frequency per mutation category calculated as each mutation’s total frequency divided by the total number of cells captured at each time point. The shaded area represents the 95% CI. Data are pooled from 4 mice per time point and 9 mice for the 70-day time point. (**I**) As in G, but showing accumulation over time of selected individual replacements.

Using the DMS data, we first sought to assess how efficiently chIgY GCs identified and selected for each of the available affinity-enhancing amino acid replacements. Because the observed frequency of a replacement in the population depends on both its Δaffinity and on the intrinsic mutability of its codon given SHM targeting biases^32,33^, we first measured nucleotide mutability across each of the *Ig*^chIgY^ alleles in the absence of selection. We engineered chIgY alleles (*Igh*^chIgY^* and *Igk*^chIgY^*) containing frameshifts in the leader sequence upstream of each V-region (Fig. S1A). The resulting alleles, while non-functional, were still transcribed and hypermutated as “passengers” in GC B cells (which use their intact WT *Ig* alleles to respond to antigen). Such passenger alleles can be used to read out the intrinsic mutability of an Ig sequence in the absence of antigen-driven selection^32^. To make these measurements in a large population of GC B cells, we first generated large GCs in *Igh*^chIgY*/WT^ and *Igk*^chIgY*/WT^ mice by infection with *Plasmodium chabaudi*^34^. We then bulk-sequenced the *Igh* and *Igk* genes of sorted pools of 7.5 x 10^5^ GC B cells at days 20-21 post-infection, when B cells carried on average 1-2 nucleotide mutations per chain. Fig. 3A shows the relative mutation rate of each nucleotide in the *Igh*^chIgY^* and *Igk*^chIgY^* sequences obtained using this system. As expected, mutability varied greatly across each sequence and was generally higher in CDRs compared to framework (Fig. 3A). Observed mutation rates correlated significantly but not perfectly with those predicted using a previously developed five-mer context model^35^ (Fig. S4B).

Intrinsic nucleotide mutability was a much stronger predictor of the frequency of amino acid replacements in GCs *in vivo* than the Δaffinity associated with each replacement (Spearman ρ = 0.67 vs. 0.22, respectively; Fig. 3B,C). Nevertheless, replacements that improved affinity (blue in Fig. 3B) were clearly enriched above the Poisson regression line, whereas deleterious replacements (in red) were depleted. To quantify this, we plotted the observed frequency of a replacement against its predicted frequency based on intrinsic mutability, defining “replacement enrichment” as the log_10_ fold-change over the regression line. Replacement enrichment was better correlated with Δaffinity (Spearman ρ = 0.46) than observed frequency alone (Fig. 3C,D) and also correlated well with Δexpression (Spearman ρ = 0.53; Fig. 3E). The correlations between replacement enrichment and both Δaffinity and Δexpression were distinctly biphasic, starting out relatively flat but steepening markedly as they approached zero for both parameters (Fig. 3D,E). Replacement enrichment responded robustly to changes in affinity and expression only above minimal “breakpoint” values. This was confirmed using segmented regression (Fig. S4C), which revealed that, in the range surrounding neutrality, a 10-fold change in affinity or expression led to on average 15- and 100-fold (1.2 and 2.0 log_10_) changes in replacement enrichment, respectively. Thus, when replacements are analyzed in aggregate, GCs appear highly responsive to even small phenotypic changes around neutral phenotypic values, but counterselection is near maximal already at moderate expression or affinity loss. Of note, although replacements causing loss of expression also tended to reduce affinity, replacements that impaired both parameters were more strongly penalized than those affecting affinity alone (specifically, within the same range of affinity reduction, replacements were more likely to be counterselected if they also caused loss of expression; Fig. S4D).

To determine how efficiently GCs identified and selected for the full complement of affinity-enhancing amino acid replacements available to them, we collected the 15 highest-affinity B cells from each replay time point (allowing only one cell per GC) and analyzed their acquisition of the full set of 104 replacements leading to an affinity gain of at least 0.4 log_10_ (Fig. 3F). GC B cells largely failed to identify affinity-enhancing amino acid replacements that required more than a single nucleotide mutation in the same codon. Only one of such 64 replacements (S109LK, made independently by two cells and located within the highly mutable CDRL3 region) was observed in the high-affinity B cell sample (Fig. 3F), and more generally, only 156 (1.8%) of 8,744 B cells sequenced in the replay experiment carried any of these 64 replacements. Low intrinsic mutability also prevented GC B cells from finding several of the beneficial replacements accessible with single-nucleotide mutations. These included positions 27, 80, and 118 in IgH and 27 and 50 in Igκ (Fig. 3F). To investigate whether these replacements might be selected for if given enough time, we immunized as in the replay experiment but using chicken IgY in alhydrogel adjuvant, which generates longer-lived GC responses^36^, then sequenced GC B cells pooled from entire LNs 10 weeks later. B cells from these mice were highly mutated (median 18.5 nt mutations, range 11-37), and included cells with very high predicted affinities (Δaffinity >4.0), indicative of prolonged GC selection. Nevertheless, no amino acid replacements with low intrinsic mutability or requiring >1 nucleotide mutation were detected among the highest-affinity B cells from each 70-dpi sample; rather, day 70 cells achieved high affinities primarily through combinations of mutations that were found in the earlier time points.

To explore these trends systematically, we generated an independent dataset consisting of a time-course of chIgY GC B cells obtained from mice immunized as in the replay experiment but sorted in bulk (i.e. B cells from multiple GCs were pooled from whole LNs of several mice per time point). Sorted cells were analyzed by droplet-based sequencing at 5, 8, 11, 14, 17, 20, and 70 dpi (Fig. S4E,F). We then used passenger allele and DMS data to categorize all accessible amino acid replacements based on mutability (low (<25%ile), medium (25-75%ile), and high (>75%ile)) and Δaffinity (negative (<−0.3), neutral (−0.3 to 0.3), positive (>0.3)), respectively (Fig. 3G). Plotting the change in frequency of replacements in each category over the time-course (Fig. 3H) yielded results in line with our previous analysis. Low-mutability replacements were largely ignored by GCs, even when they led to substantial gains in affinity, with beneficial but unlikely replacements failing to accumulate even at 70 dpi. On the other hand, enrichment for affinity-enhancing replacements was evident in the high and intermediate mutability classes, becoming noticeable already at 8 dpi for the former and 11 dpi for the latter. Replacements in the high-mutability category accumulated progressively over time even if they were neutral (but not if they were deleterious) with respect to affinity, again indicating strict counterselection of affinity-reducing replacements. Following the accumulation of selected replacements over time revealed a pattern where neutral but high-mutability replacements (such as the S to N changes in positions S57_H_, S64_H_ and S109_L_) accumulated early on, whereas unlikely but beneficial replacements (such as the changes in D28_H_ to V, A, or G) caught up only at the much later time points (Fig. 3I).

In summary, although GCs select efficiently for changes in affinity and expression, affinity maturation is heavily constrained by intrinsic biases in SHM and accessibility through single-nucleotide changes. These constraints limit the exploration of the full mutational landscape, even under prolonged selection.

### Phylogenetic analysis identifies the drivers of affinity maturation

Assigning affinity and Ig expression levels to every cell across 119 GC B cell phylogenies enables us to compare different evolutionary strategies—such as clonal bursting versus the parallel evolution of multiple lineages—in terms of their effectiveness in generating high-affinity B cell populations. Below we describe key findings emerging from this analysis.

#### GCs select consistently for increases in affinity and maintenance of Ig expression

**Figure 4.**
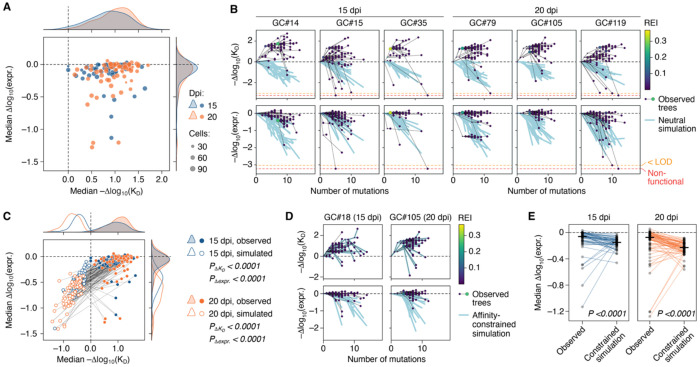
Quantifying germinal center selection for affinity and for maintenance of Ig expression. (**A**) Distribution of 119 replicated GCs by median affinity and Ig expression, as quantified using the additive DMS model. Each symbol represents one GC, symbol sizes are proportional to the number of cells sequenced. Densities on the top and right show the distribution of GCs according to Δaffinity and Δexpression, respectively. (**B**) Example trajectory plots in which phylogenetic trees are plotted in 2D space according to their number of somatic mutations and Δaffinity (top) or Δexpression (bottom). For the experimentally observed trees, circles representing individual nodes, colored by REI and scaled by number of identical sequences, are connected by black lines. Simulated trees with the same phylogenetic structure but in which mutations are assigned based on mutability alone are shown as light blue lines, which represent the median values for each node in 10 simulated trees. Plots for all GCs available at https://github.com/matsengrp/gcreplay/tree/main/results/notebooks/phenotype-trajectories/naive_reversions_first. (**C**) Distribution of 119 replicated GCs by median affinity and Ig expression as in (A) (filled symbols), paired to the medians of 10 simulated GCs as in (B) (open symbols). P-values are for the Wilcoxon signed-rank test. (**D**) Example trajectories as in (B), but simulations are constrained by affinity—i.e., replacements are assigned based on nucleotide mutability but must match the Δaffinity of the observed node within 0.05 log_10_(ΔK_D_). (**E**) Comparison of median Δexpression between experimental GCs and affinity-constrained simulations as in (D). Each symbol represents one experimental GC (“observed”) or the median of 10 simulated GCs (“simulated”).

Whereas GC phylogenies varied widely in structure (Fig. 1D,F and Fig. S2), replay GCs were much more consistent with respect to affinity maturation. Median affinity was higher than that of the unmutated ancestor in 117 of 119 GCs sampled, while variance among GCs was relatively low (Δaffinity = 0.87 ± 0.38 at 15 dpi and 1.00 ± 0.34 at 20 dpi (mean ±SD); Figs. 4A and S5A). The consistency of selection for affinity and expression across GCs was more evident when the observed trees were displayed alongside neutral drift simulations in mutational load vs. Δaffinity “trajectory plots” (Fig. 4B). In neutral drift simulations, replacements are introduced according to mutability alone, in the absence of affinity-based selection, while maintaining the phylogenetic structure of each GC. These simulations yielded median affinities that were consistently well below that of the naïve ancestor (Δaffinity = −0.47 ± 0.24 at 15 dpi and −0.70 ± 0.26 (mean ±SD)) and markedly lower than those observed experimentally (Fig. 4C). Thus, despite the strong downward pressure on affinity exerted by stochastic mutagenesis, GCs consistently achieve increases in affinity over time.

In contrast to affinity, SHM led to a noticeable decrease in Ig surface expression, consistent with previous observations^37^. Median Δexpression dropped below the naïve starting point in 106 of 119 GCs (Δexpression = −0.13 ± 0.21 at 15 dpi and −0.17 ± 0.27 at 20 dpi (mean ±SD)), with several instances of more substantial losses (Fig. 4A–C and S5A). The four GCs in which median Δexpression fell below −1.0 (Fig. 4A and S5B) were particularly informative. In all four GCs, the median-expression B cell carried the replacement Y42_L_N, which has the uncommon property of causing a severe drop in expression (Δexpression = −1.27) while still increasing antigen binding (Δaffinity = 0.26; this replacement is indicated by an arrowhead in Fig. S3D). Thus, even strongly destabilizing replacements can still undergo positive selection if they lead to gains in affinity. As with Δaffinity, Δexpression was also much lower in simulated trees than in experimental ones (Δexpression = −0.48 ± 0.22 at 15 dpi and −0.73 ± 0.25 at 20 dpi (mean ±SD); Fig. 4C). Because affinity and expression are partially correlated (Fig. 2B), these data do not allow us to determine whether GCs were selecting specifically for maintenance of Ig expression. To investigate this directly, we performed additional tree-based simulations in which we forced simulated trees to match the Δaffinity of experimental GC lineages within 0.05 log_10_(K_D_), regardless of the effects of simulated replacements on Δexpression. Under these conditions, independent selection for Ig stability would result in higher median Δexpression in experimental GCs compared to their simulated counterparts. Indeed, median Δexpression remained marginally higher *in vivo* than in simulations (medians, −0.060 vs. −0.15, at 15 dpi and −0.073 vs. −0.23 at 20 dpi, p < 0.0001 for both comparisons; Fig. 4D,E), indicating some degree of selection acting specifically on Ig expression.

Taken together, our results show that, despite wide variation in tree shapes, GCs reliably and efficiently select for increased antibody affinity, even in the face of the downward pressure of random mutation. Although, GCs appear to exert some pressure to maintain Ig expression levels independently of selection on affinity, even large losses in expression can be tolerated if compensated by affinity gains.

#### Low-affinity B cell lineages are efficiently terminated

**Figure 5.**
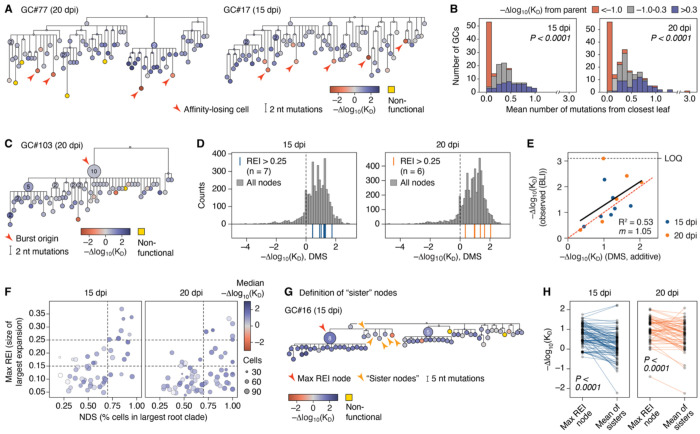
Drivers of affinity maturation as determined by phylogenetic analysis. (**A**) Example phylogenies colored by Δaffinity, indicating cells that lost substantial affinity and are located at terminal nodes (red arrowheads). (**B**) Observed and inferred nodes from each replay GC phylogeny were divided into those that lost (<−1.0), maintained, (−1.0 to 0.3) or gained (>0.3) affinity with respect to their parent node. For each GC, the mean distance between the nodes in each category and the nearest leaf was then recorded (a distance of 0 indicates that the node is itself a leaf). The plot shows the distribution of mean values for each category in each GC. (**C**) Example of a clonal burst phylogeny colored by Δaffinity. The burst point is indicated by a red arrowhead. (**D**) Distribution of Δaffinities for clonal burst nodes (REI >0.25; blue lines for 15 dpi and orange lines for 20 dpi) compared to the distribution of Δaffinities for all nodes (observed and inferred) from the same time point (grey bars). (**E**) Comparison of Δaffinities predicted using the additive DMS model and BLI measurements of Fabs produced recombinantly based on the *Ig* sequences of each of the bursting nodes in (D). LOQ, limit of quantitation. Solid black line is the linear trend (excluding the Fab above the LOQ); dotted red line is *x = y*. (**F**) Distribution of NDS and max REI scores for GCs from 15 and 20 dpi as in Fig. 1E and F but colored by median Δaffinity; each symbol represents one GC scaled according to the number of B cells sequenced. (**G**) Schematic explaining the definition of “sister” nodes used in (H) and Fig. S5D. (**H**) Comparison of the affinity of the max REI node in each replay GC and the mean affinity of its “sister” nodes, as defined in Fig. 5G. P-values are for the Wilcoxon signed-rank test.

A prominent feature in GC trajectory plots is the observation that steep downward-trending lines almost always lead to terminal nodes that lack detectable descendants (for example, GCs #15 and #119 in Fig. 4B). The same feature was evident in GC phylogenies colored by affinity, where affinity-losing B cells appeared predominantly as red-tinted “leaves” (indicated by arrowheads) at the terminal ends of branches, downstream of higher-affinity (blue-tinted) internal nodes (Fig. 5A and Fig. S2). This pattern suggests that GC B cells that lose substantial affinity, rather than continuing to proliferate and evolve, are instead efficiently terminated. To quantify this observation, we calculated the Δaffinity from the node of interest to its immediate ancestor (which approximates how much affinity a node gained or lost in its most recent round of SHM) divided into three categories: <−1.0, −1.0 to 0.3, and >0.3. We then plotted, for each GC, the mean mutational distance between nodes in each category and the closest branch tip (a proxy for how recently these mutations took place). This showed that, at both time points, nodes that lost affinity compared to their immediate ancestors were disproportionately enriched at leaves (i.e., more likely to be at distance zero from the closest branch tip, and thus to have been recently generated; Fig. 5B). We conclude that B cells that lose substantial affinity due to SHM rarely persist in GCs. By extension, the low-affinity B cells observed in our dataset likely represent recent mutants not yet purged from the population by selection.

#### Clonal bursts are not major drivers of affinity maturation

Clonal bursts represent extreme jackpot events in which a single B cell proliferates enough to eliminate most or all competing lineages in its GC of origin^10,38^. If burst size is reliably determined by affinity, sporadic large bursts could act as evolutionary leaps, driving the average affinity of the population upwards in a stepwise manner. Counter to this notion, the largest clonal bursts found at 15 and 20 dpi (Fig. 1E,F, top-right sector) originated from B cells that, despite having relatively high affinity, were far from being extreme outliers (Fig 5C,D; specifically, the mean percentile rank of bursting nodes within the affinity distributions of their respective time points was 66 (range, 30–94) at 15 dpi and 64 (range, 21–97) at 20 dpi). We verified that these observations were not due to errors in the additive DMS model by measuring the affinities of recombinant Fabs derived from all 13 clonal bursts by BLI. With the exception of one Fab whose affinity fell outside the range of the instrument, clonal burst affinities were relatively well predicted by the DMS (R^2^ = 0.53, slope = 1.05), with most predictions laying either slightly above or slightly below the *x* = *y* line (Fig. 5E). Thus, although clonal bursts derive from B cells with affinities that are somewhat higher than average, this advantage is not compatible with a role for bursts as exclusive or even major drivers of affinity maturation at the population level. This pattern also implies that clonal bursting may not be a superior strategy for affinity maturation when compared to more gradual evolution. Accordingly, color-coding GCs in the NDS x max REI scatter plot (Fig. 1E,F) by median Δaffinity revealed no marked enrichment for high-affinity cells in the upper right “clonal burst” sector (Fig. 5F). Similarly, there was no strong correlation between affinity measures and burst size (REI). Of note, however, Δaffinity correlated moderately well with how dominated a GC was by its largest lineage (NDS), especially at the day 15 time point (Fig. S5C). Thus, evolving multiple, relatively equal lineages simultaneously is generally associated with lesser affinity gains. Nevertheless, clonal bursts are neither the primary drivers of affinity maturation nor inherently superior to less punctuated evolution as a strategy to increase affinity within an individual GC.

#### Weak but consistent selection yields reproducible affinity gains

Given the absence of a strong link between clonal bursting and affinity gain, we sought to identify common drivers of affinity maturation present in all GCs, regardless of large-scale tree topology. To this end, we selected the highest-REI B cell in each GC as well as all of its “sister” nodes within the same branch (nodes that shared a common immediate ancestor with the node of interest), which we reasoned would be representative of the competitors of the highest-REI B cell at the time it was selected (Fig. 5G). We then measured the gain in affinity between each node and its sisters and that of their immediate ancestor. This showed that, although the discrimination between bursts and sisters was imperfect (i.e., the highest-REI node did not always have higher affinity than its sisters), it was on aggregate significantly skewed towards expanding higher-affinity B cells over their lower-affinity competitors (Fig. 5H and S5D).

Taken together, our data indicate that affinity maturation in GCs, rather than being driven by sporadic clonal bursts, results from persistent selection that is relatively inaccurate but sufficiently biased towards high affinity B cells to reliably favor their expansion over time.

### Phylodynamic survivorship bias distorts tree-based signals of GC B cell fitness

**Figure 6.**
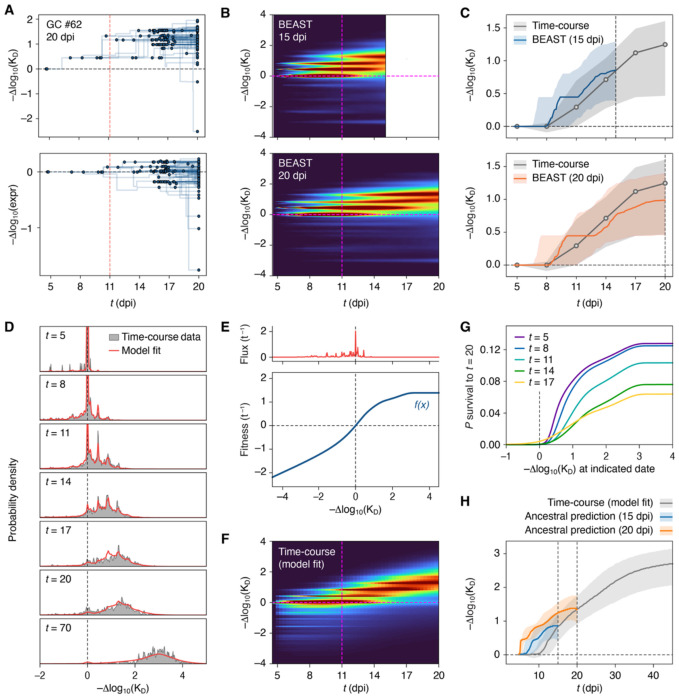
The reconstructed evolutionary process and affinity-fitness landscape. (**A**) Example time-resolved tree for one GC showing nodes (birth events) and mutations (affinity jumps) in time (dpi), with all leaves at the sampling time (20 dpi for this GC). 100 such candidate trees were sampled for each GC. Taking a time slice from one such tree (e.g., at the red dotted line) yields a collection of ancestral cells and their associated affinities. (**B**) Heatmaps showing the evolution of affinities over time resulting from aggregating all candidate time-resolved trees for 15 and 20 dpi GCs. Dotted lines given for reference. The push of the past and pull of the present are seen as excess densities in the upper-left and lower-right quadrants, respectively, compared to (F). (**C**) Grey trendline shows median and interquartile range (IQR) affinity in the time-course experiment at 5, 8, 11, 14, 17, and 20 dpi. The blue and orange trend lines show median and IQR affinity through time derived from time-resolved trees for 15 dpi and 20 dpi GCs, respectively. (**D**) Distributions of affinity at 5, 8, 11, 14, 17, and 20 dpi in the time-course experiment (grey) compared to time-slice fits using the fitness landscape model (red). (**E**) Key parameters driving the fitness landscape model: the distribution of affinity mutations specified by DMS effects and mutation propensities (upper panel), and the fitness landscape (lower panel). (**F**) Solution of the fitness landscape model showing the affinity distribution evolving in continuous time up to day 20 for comparison with (B) and (C). Dotted lines given for reference. (**G**) Probabilities of survival until 20 dpi for cells of different affinity at various previous times, given the fitted fitness landscape model. These curves are interpreted as the distortions that elevate or suppress different affinities in the reconstructed process. (**H**) The solution *p(x, t)* of the fitness landscape model (grey) summarized as median and IQR affinity evolving in continuous time. Orange and blue show predicted median and IQR affinity for ancestral population histories sampled at 15 dpi and 20 dpi, respectively, by reweighting the solution with survival probabilities as shown in (G).

A prominent feature of GC trajectory plots was that many trees appeared to make substantial gains in affinity with their first few mutations followed by a plateau and an eventual decay of affinity at the leaves, suggesting that GC selection quickly slows down after making initial progress (Fig. 4B). To measure these temporal trends over the ensemble of all trees, we first used Bayesian phylogenetics^39^ to generate time-resolved phylogenies for each GC (see Methods). These time-resolved trees (as exemplified in Fig. 6A) represent branch lengths as inferred time intervals, and place mutations and nodes at concrete times in the history of the GC prior to sampling. Because time-resolved tree details have considerable statistical uncertainty, we used 100 posterior tree samples (each a candidate time-resolved tree) for each GC. We then used these time-resolved trees to reconstruct the evolution of B cell affinity over time across all GCs in the replay experiment (Fig. 6B,C). This analysis confirmed the observations made from individual trees: affinity-increasing mutations appear to emerge early and then be maintained for much of the GC reaction as affinity plateaus (upper-left quadrant in Fig. 6B), followed by the emergence of a minor subset of lower-affinity B cells at sampling time (lower right). Therefore, at face value, tree-based reconstruction of GC dynamics is suggestive of rapid changes in the strength of affinity-based selection early in the reaction.

To assess if these dynamical features were reproduced in a true time-series, we assigned affinities to all B cells in the time-course experiment (Fig. S4E,F), which sampled pooled B cells from whole LNs (and thus, dozens of GCs combined) at discrete time points (Fig. S6). Evolution of affinity in this time-course differed markedly from that inferred based on the tree-based reconstruction (Fig. 6C). First, affinity was found to increase gradually through day 17, rather than making early gains followed by a longer plateau. Second, there were many more negative affinity cells at early time points compared to days 14-20 of the time-course (Fig. S6A,B). Thus, phylodynamic reconstruction based on an extant population fails to predict the true progression GC B cell affinities over time.

We hypothesized that this inconsistency could be explained by two concepts first described in the macroevolution literature: the “push of the past” and the “pull of the present”^40^. The evolutionary process reconstructed based on a phylogeny—even if inferred perfectly—inevitably represents a biased history of the sub-population of organisms or cells destined to become the ancestors of those sampled at the endpoint. Thus, by looking backwards in time, we are biased towards observing the fitter subpopulation that survived to sampling, rather than the entire distribution of affinities present at the reconstructed time point. This survivorship bias is referred to as the “push of the past.” Conversely, the apparent late emergence of low-affinity B cells and the absence of such cells from reconstructed early time points may stem from the “pull of the present”: low-affinity cells are only observed in the tree near the sampling time, because selection has not yet purged these cells from the population.

To test this interpretation, we began by developing a minimal mathematical model^41–44^ for how the distribution of affinities evolves through time. Rather than focusing on individual mutations or cells, our model predicts the overall distribution of binding affinities *x* (measured as log_10_ affinity change from naïve) across the GC population through time *t*. We denote this distribution *p*(*x, t*) and fit the model using the time-course experiment data (Fig. 6D). The model incorporates two key biological effects: (1) random mutations on antibody affinity, based on our deep mutational scanning and passenger mouse experiments (Fig. 6E, upper panel); and (2) a fitness landscape *f*(*x*) that determines growth rate based on affinity *x* (Fig. 6E, lower panel). The landscape *f*(*x*) quantifies the outcomes of affinity-based competition: the fitness landscape of clone 2.1 is approximately linear over most of the affinity range but shows clear saturation beginning at about 2 orders of magnitude above the naïve affinity. Above about 3 orders of magnitude of affinity increase (~40 pM), there is essentially no marginal response to further affinity gains, possibly reflecting a previously described “ceiling”^45,46^ beyond which GCs cannot sense further gains in affinities. Fig. 6F presents the full solution continuous in the time domain, shown up to 20 dpi for comparison to the tree-based plots in Fig. 6B. This continuous solution confirmed the differences between tree-based histories and time-course measurements shown in Fig. 6C).

We then used this fitted model to predict the “push of the past” and “pull of the present” distortions that should be expected in trees where we are only able to see the sub-distribution of *p*(*x, t*) that is destined to be ancestral to a future sample. Using a forward master equation approach (see Methods), we computed the probability of stochastic extinction of a lineage prior to sampling time at 15 or 20 dpi. Example functions for 20 dpi are shown in Fig. 6G, and the predicted affinity distribution of surviving lineages only are shown in Fig. 6H, alongside the full population MLE. These predictions show clear distortions—early jumps of affinity followed by subsequent slowing down before the sampling time—that are qualitatively consistent with our findings from inferred trees (Fig. 6C), indicating that these features of reconstructed trees indeed represent push-of-the-past and pull-of-the-present distortions related to survivorship bias.

We conclude that both sampling of current affinities and phylogenetic reconstruction of past trajectories from extant GC B cell populations are subject to survivorship biases that can distort our view of selection dynamics. Nonetheless, modeling these effects allows us to separate true biological processes from observational artifacts, providing insight into how mutation and selection shape antibody evolution.

## DISCUSSION

We present the results of an experimental evolution system in which we replay a simplified, monoclonal GC reaction over one hundred times. Our primary goal was to gain insight into the extent to which various features of GC selection can be viewed as “reproducible”—that is, as producing common outcomes over multiple runs. We find that, even in this simplified setting, GC selection yields widely divergent tree topologies, from clonal burst-type structures consisting almost fully of descendants of a single B cell to multi-pronged GCs in which several lineages evolve in parallel. Thus, the diversity of GC outcomes observed in our previous work using Brainbow GCs^10^ cannot be attributed exclusively to differences in founder populations in a polyclonal setting. By contrast, DMS-based affinity estimation showed that phenotypic evolution across GCs is broadly consistent. Regardless of tree shape, virtually all GCs gained affinity compared to the naïve ancestor, which is all the more remarkable given that selection happens over a landscape that overwhelmingly favors deleterious replacements. Moreover, when accounting for intrinsic mutability, GC selection consistently enriches for amino acid replacements that lead even to very small gains in affinity. Thus, GCs are reproducible with respect to phenotype if not to phylogeny.

A simple additive model in which the log_10_ DMS-measured effects of single replacements are added, ignoring non-additive epistatic interactions, was sufficient to predict the affinities of clone 2.1 variants carrying multiple replacements with a degree of accuracy sufficient to resolve large-scale features of GC selection. This observation agrees with a recent study of several type-specific SARS-CoV-2 antibody lineages that found epistatic effects to be infrequent^47^. On the other hand, studies of broadly neutralizing antibodies (bnAbs) against influenza show more substantive epistatic effects^48,49^. A reason for this discrepancy may lie in the specific structural features clone 2.1—as noted, most affinity-enhancing replacements were spread out along the edges of an otherwise optimal paratope (Fig. 2F), making them less likely to interact with each other than the clustered replacements found in influenza bnAbs^48,49^. Another factor may be the relatively sparse nature of the evolution of high affinity in clone 2.1, as only 3-4 affinity-enhancing replacements were needed for a B cell to reach the top stratum of affinity by 20 dpi (Fig. 3F). This was also found to be a feature of type-specific SARS-CoV-2 antibodies^47^, but is in contrast to the more convoluted evolutionary pathways required for broad neutralization^48,49^. The relative absence of epistasis among affinity-enhancing replacements in clone 2.1 may partly explain why GCs are reproducible at the phenotypic but not at the phylogenetic level. Because the effects of replacements are largely additive, many combinations of mutations, made in any order, can lead to similar gains in affinity. This avoids the need to follow particular mutational trajectories, as may be required for bnAb generation^48,49^. Despite their overall usefulness, additive DMS predictions were occasionally inaccurate, as exemplified by the marked underestimation of the affinity of one of the clonal bursts in Fig. 5E. Thus, conclusions based on single or few antibody sequences—such as the finding that clonal bursting is not restricted to the highest-affinity B cells—must be validated through direct biochemical measurements, as we have done here. On the other hand, errors in DMS are likely to average out across large populations of cells, so that aggregate predictions are likely sufficiently accurate not to require further validation.

The access to the full set of affinity-enhancing replacements available to clone 2.1 granted by DMS revealed that GCs in fact “miss” the large majority of replacements with affinity-enhancing potential. This substantial blind spot was related primarily to codon constraints—i.e., GCs are virtually unable to select for amino acid replacements that require two nucleotide mutations to the same codon, even after extended periods of affinity maturation. The low intrinsic mutability of many *Ig* positions due to AID and SHM targeting biases^32,33^ further suppresses acquisition of affinity-enhancing replacements, even among those accessible by a single mutation. Inaccessibility along these lines has been previously shown to be a roadblock to the formation of certain bnAbs to HIV, and the need to acquire such improbable replacements may partly explain why HIV bnAbs are so extensively mutated^50,51^. Our data allow us to quantify the full extent of these biases; they also suggest that DMS may be used to increase the affinities of monoclonal antibodies well beyond what is obtainable by affinity maturation *in vivo*.

A number of previous studies reported relatively high frequencies of low-affinity B cells within GCs, including both low-affinity members of otherwise high-affinity B cell clones and entire clones composed of low-affinity B cells^10–13,52–54^. These observations raised the possibility that GCs might be permissive to the presence of low-affinity B cells, either as a strategy to help lineages to traverse low-affinity “valleys” on the way to higher-affinity configurations or simply as a means to maintain broader clonal diversity. Replaying GCs across a quantified affinity showed that, instead, most affinity-losing B cells in a GC fail to leave detectable descendants. Thus, low-affinity outliers within high-affinity clones likely represent recent loss-of-function mutants that have not yet been purged by selection (a notion substantiated by our subsequent pull-of-the-present analysis), suggesting very low tolerance for loss of affinity in the GC. How then can the presence in GCs of entire clones of B cells with very low or undetectable affinities for antigen be accounted for? A speculative explanation is that biochemically measured affinities may not be predictive of a B cell’s ability to retrieve antigen from FDCs *in vivo* when different clones specific for different epitopes are compared. For instance, B cell clones with low measured affinities may recognize cryptic “dark” epitopes that are hidden in biochemical assays but become exposed when antigen is partly degraded or otherwise distorted on the FDC surface^11,55^. Alternatively, circulating antibodies may block access to antigen by immunodominant B cell clones^53,56–58^, confounding the relationship between a B cell’s nominal affinity and its capacity to retrieve antigen *in vivo*^59^. A testable prediction of this model is that competing B cell clones with vastly different measured affinities may actually be similar in their ability to retrieve antigen and present it to Tfh cells.

Likewise, the finding that clonal bursts virtually eliminate clonal diversity in some GCs while others remain highly polyclonal^10^ led us to speculate previously that bursting may represent a trade-off where diversity is exchanged for the ability to expand “winner” clones with exceptionally high affinity^60^. Our phylogenetic analyses again fail to support such a model, at least for this particular clone (see *Limitations*, below). First, clonal bursts originated from B cells spanning the whole spectrum of affinities present in GCs at the time of sampling, from near germline to beyond the range of our BLI assay. Second, GCs containing large bursts did not have higher median affinities than those lacking such expansions. Thus, a scenario in which affinity maturation is the result of sporadic large-scale expansion of very high-affinity clones is inconsistent with our observations. On the other hand, comparing the most successful node of each GC to its sisters—a proxy for the competition that particular node was facing at the time of its decision to expand—revealed a distinct if imperfect association between affinity and number of progeny. Thus, while selection at the level of individual B cell fate decisions may appear noisy^10,38^—allowing clonal bursts to occur apparently independently of affinity, especially when analyzed in small numbers—this same process reliably drives long-term affinity maturation when repeated across thousands of individual decisions. The multi-step process by which competition for T cell help drives GC selection^9,61^ offers ample opportunity for stochasticity to influence the outcomes of selection. These include the mapping of BCR affinity onto peptide-MHC density (i.e., cells with higher affinity may not always be the ones to acquire and present the most antigen), the local nature of B cell competition for T cells (i.e., each T cell evaluates only the B cells in its immediate neighborhood), and heterogeneity in affinity and fitness among T cells themselves^62,63^. The fact that clonal bursting is exponential serves to amplify these small stochastic differences^10,38^, also providing an explanation for why clonal bursts do not always originate from the highest-affinity B cells. Thus, GCs may be viewed as “imperfect cell sorters,” which decide on the fate of B cells with a relatively low degree of accuracy but with sufficient bias to, in the long run, improve affinity deterministically.

Lastly, our fitness-based modeling shows that both direct sampling of GC B cells and reconstruction of their ancestral histories are subject to systematic biases that can obscure the true dynamics of selection, as proposed initially in the context of macroevolution^40^. Our modeling confirms that the presence of low-affinity cells near the time of sampling does not imply that GCs broadly tolerate such cells—rather, it reflects a pull-of-the-present effect whereby such cells are observed simply because they have not yet been eliminated. Conversely, the early appearance of high-affinity lineages in reconstructed trees arises from the push-of-the-past effect—a survivorship bias that leads to the overrepresentation in historical accounts of lineages that happened to leave descendants. These sampling biases create the misleading impression that selection strength is initially strong but quickly weakens, even in a population that is evolving at a constant rate. By modeling the selection process explicitly, we can correct for such distortions and better understand how mutation and selection act in real time to drive the maturation of antibody affinity.

### Limitations of the study

Our study traces the outcomes of GC selection acting on one particular B cell clone (2.1) responding to a single model antigen (IgY). The original affinity of clone 2.1 for IgY is relatively high (40 nM) but is representative of the naïve affinities of successful clones that come to dominate secondary responses in mice^64^. Nevertheless, it is possible, and even likely, that particular features of GC evolution will differ in clones with lower starting affinities. For example, lower-affinity clones may have single amino acid replacements that confer more than the <10-fold gains in affinity available to 2.1; these replacements could provide affinity jumps that are large enough to drive clonal bursting in a more deterministic fashion. Further experimentation with different antigen-antibody pairs may help resolve such issues.

## MATERIALS AND METHODS

### Laboratory Methods

#### Mice

*Igh*^2.1^ and *Igk*^2.1^ mice were generated by CRISPR-Cas9 gene-editing in fertilized mouse oocytes. For *Igh*^2.1^, we inserted the pre-rearranged unmutated V_H_ sequence of clone 2.1, preceded by the 443 bp proximal promoter of *Ighv9-4*^65^. in place of the 4 endogenous *Igh* J segments, using two flanking sgRNAs and a single-stranded DNA (ssDNA) megamer (IDT) as described in the easi-CRISPR method^66^. *Igk*^2.1^ mice were generated by inserting the pre-rearranged unmutated V_κ_ sequence of clone 2.1, preceded by the 188 bp proximal promoter of *Igkv3.12*^65^, in place of the 5 endogenous *Igk* J segments, using two sgRNAs and an ssDNA template generated by PCR amplification of a cloned template plasmid followed by opposite-strand digestion using the Guide-it Long ssDNA production system v2 (Takara Bio). Passenger allele mice (*Igh*^2.1^* and *Igk*^2.1^*) were generated using single sgRNAs to introduce indels into the leader sequence of fertilized *Igh*^2.1/+^ and *Igk*^2.1/+^ oocytes, respectively. Sequences of the sgRNA protospacers and ssDNA donor templates used for mouse generation are provided in Supplemental Spreadsheet 2). CD23-Cre (Fcer2a-Cre) BAC transgenic mice^20^ (Jax strain #028197) were provided by M. Busslinger (IMP Vienna). *Bcl6*^f/f^ mice^21^ (Jax strain #023727) were provided by A. Dent (U. Indiana). PAGFP-transgenic mice^9^ (Jax strain #022486) were generated and maintained in our laboratory. All animal experiments were approved by the Rockefeller University’s Institutional Animal Care and Use Committee (IACUC).

#### Cell transfers, immunizations, and Plasmodium infection

Resting splenic B cells were purified by filtering splenocytes through a 70 μm mesh into PBS supplemented with 0.5% BSA and 1mM EDTA (PBE). CD43 MACS beads were used to purify resting B cells from single-cell suspensions according to the manufacturer’s protocol (Miltenyi Biotec). The percentage of *Igh*^2.1/+^/*Igk*^2.1/+^ resting B cells was determined prior to transfer by staining with chicken IgY-BV421 (conjugated in-house) followed by flow cytometry. For all experiments, 5 x 10^5^ *Igh*^2.1/+^/*Igk*^2.1/+^ resting B cells were transferred into each recipient mouse intravenously. 24 hours following cell transfer, mice were immunized subcutaneously in the hind footpads, inner thighs, and/or forearms with 5, 10, and 20 μg, respectively, of chicken IgY (Exalpha Biologicals) precipitated in 1/3 volume of Imject Alum (ThermoFisher Scientific Cat# 77161) to generate similar-sized GCs in popliteal, inguinal, brachial, and axillary nodes. The immunization schemes were the same for the day-70 experiments, except that Imject Alum was replaced with Alhydrogel (InvivoGen) used according to the manufacturer’s protocol. For analysis of SHM in passenger alleles, either *Igh*^2.1^*^/+^.*Igk*^+/+^ or *Igh*^+/+^.*Igh*^2.1^*^/+^ mice were injected intravenously with 10^5^ *Plasmodium chabaudi*-infected red blood cells (BEI Resources Cat# MRA-741).

#### Imaging and Photoactivation

Multiphoton imaging and photoactivation were performed as described previously^9,10^, using an Olympus FV1000 upright microscope fitted with a 25X 1.05NA Plan water-immersion objective and a Mai-Tai DeepSee Ti-Sapphire laser (Spectraphysics). One day prior to photoactivation, PAGFP-transgenic mice were injected intravenously with 10 μg of a non-blocking antibody to CD35 (clone 8C12, produced in house) conjugated to Cy3 to label networks of follicular dendritic cells (FDCs)^67^. A Leica M165FC fluorescence stereomicroscope with a dsRed filter was used to register the location of FDCs. Clusters of CD35-expressing cells were then identified using multiphoton imaging at λ = 950 nm, at which photoactivation does not take place, and three-dimensional regions of interest were photoactivated by higher-power scanning at λ = 830 nm. Lymph nodes were then sliced manually under a Leica M165FC fluorescence stereomicroscope using double-edged safety razor blades (Astra Superior Platinum) for subsequent flow cytometry and sorting.

#### Flow cytometry and cell sorting

For replay time points, sliced LN fragments (15 and 20 dpi) or whole LNs (70 dpi) were placed into microcentrifuge tubes containing 100 μl PBE, macerated using disposable micropestles, and dissociated into single-cell suspensions by gentle vortexing. We then added 100 μl of 2× antibody stain (B220-BV785, TCRβ-APC Cy7, CD38-APC, Fas-PE-Cy7, biotinylated chicken IgY, Streptavidin-BV421, mouse Igκ-PE, supplemented with Fc block) to the cell suspension, which was incubated on ice for 30 min. Photoactivated transferred GC B cells were index-sorted into 96-well plates containing 5 μl TCL buffer (Qiagen) supplemented with 1% β-mercaptoethanol. For passenger allele sequencing, spleens from *P. chabaudi*-infected mice were harvested at 20-21 days post-infection. Single-splenocyte suspensions were obtained by forcing spleens through a 70 μm mesh followed by hypotonic lysis of red blood cells using ACK buffer. CD19 magnetic (MACS) beads (Miltenyi Biotec) were used according to the manufacturer’s protocol to enrich for B cells prior to sorting for splenic GC B cells as described above. For 10X Genomics single-cell *Ig* sequencing, LN cell suspensions were stained with individual hashtag oligonucleotide (HTO)-labeled antibodies to CD45 and MHC-I for sample barcoding prior to GC B cell sorting as above. A combination of two HTO’s per sample was used to accommodate for the number of samples in the experiment. Cells were sorted into microfuge tubes with PBS supplemented with 0.4% BSA and were counted for viability by trypan blue staining prior to loading onto a 10X Genomics Chromium Controller.

#### Single-cell PCR amplification and sequencing of Igh^2.1^ and Igk^2.1^ alleles

Sorted single cells were processed and analyzed essentially as described previously^10^, except that specific forward primers were used to amplify chIgY BCR, and both chains were amplified simultaneously for all wells. Primers were limited to the leader sequences of each *Ig*^chIgY^ allele to enable full sequencing of framework (FR)1 regions. Specific primers used were 5’-AGCGACGGGAGTTCACAGACTGCAACCGGTGTACATTCC-3’ (*Igh*^2.1^ V_H_ leader forward recoded) and 5’-AGCGACGGGAGTTCACAGGTATACATGTTGCTGTGGTTGTCTG-3’ (*Igk*^2.1^ Vκ leader forward). The first 18 nucleotides of the *Igh*^2.1^ V_H_ leader forward sequence were prepended to the Vκ primer so that same barcoding primers could be used for both chains in the next step. After PCR reactions as described previously^10^, pooled PCR products were purified using SPRI beads (0.7× volume ratio), gel-purified, and sequenced with a 500-cycle Reagent Nano kit v2 for single-cell libraries on the Illumina Miseq platform.

#### Bulk PCR amplification and sequencing of Igh^2.1^* and Igk^2.1^* passenger alleles

750,000 GC B cells were sorted directly into 750ul of TRIzol LS (Thermofisher #10296010), bulk RNA was extracted using the manufacturer’s protocol. Quality of RNA was checked using TapeStation D1000, and only samples with RIN > 8 were used for generating BCR libraries. The NEBNext Immune Sequencing kit mouse (#E6330S) was used according to the manufacturer’s instructions, with IgM, IgGa, and IgGb primers used to sequence BCR heavy chains and Igκ primers for light chains. Each chain was amplified in separately to ensure even amplification. BCR libraries were sequenced using Illumina NextSeq 2000 flow cell P1, to generate 100 million reads.

#### *10X Genomics single-cell* Ig *sequencing*

GC B cells sorted as above were sequenced for gene expression and B cell receptors (BCR) using the 10x Chromium Next GEM Single Cell 5’ Reagent kit v3 with Feature Barcode technology for Cell surface Protein, according to the manufacturer’s protocol. The resulting library was sequenced on a NovaSeq SP (Illumina) flow cell with a minimum sequencing depth of 30,000 reads per cell. De-multiplexing of the samples based on their unique molecular identifier (UMI) and HTO counts were generated with Cell Ranger v6.0.1, v7.0.1 or v8.0.1 with mm11 reference. BCR libraries were also processed with CellRanger “vdj” with default parameters.

#### Recombinant Fab fragment production and affinity measurements

Fabs were produced in one of two ways. For Fig. 2H, *Igk*^chIgY^ and *Igk*^chIgY^ variable regions were synthesized by Twist Bioscience and directly cloned into a custom human IgG1 Fab expression vector, as described previously^10^. Plasmids were transfected into Freestyle Expi-293 suspension cells (Life Technologies), and monoclonal Fab fragments were purified using Ni-NTA beads (GE Healthcare), according to the manufacturer’s protocol. Protein purity was assessed by SDS-PAGE and functional protein concentrations were calibrated using biolayer interferometry on an Octet Red96 instrument using anti-Fab coated sensors (FortéBio). For Figs. 2I and 5E, Fabs were cloned and produced by GenScript based on *Igh*^chIgY^ and *Igk*^chIgY^ variable region sequences and were purified using Capture Select CH1-XL Magnetic Agarose Beads (ThermoScientific). Purity and functional concentrations were measured as mentioned above. BLI affinity measurements were performed as described previously using Octet SAX biosensors^10^. K_D_ values were calculated using a kinetic 1:1 model. Only Fabs with good global fits (R^2^ > 0.98) over multiple concentrations were used for K_d_ calculations. For Fabs that could not fit globally due to their low affinity (n = 3), partial fits were averaged over multiple concentrations and used instead.

#### Yeast-surface display deep mutational scanning

The clone 2.1 scFv was ordered as a yeast codon-optimized gene and cloned into the pETcon yeast surface-display expression vector containing a previously described barcode sequencing landing pad^68^. A site-saturation mutagenesis library was synthesized by Twist Bioscience, precisely encoding every possible amino acid substitution at each of the sites in the heavy- and light-chain variable domains. In duplicate, N16 barcodes were appended to mutagenesis products and cloned into the vector as previously described^68,69^. The duplicate libraries were electroporated into *E. coli* (NEB C3020K) and plated in to a bottlenecked target of 88,000 cfu per library, aiming for an average of 20 redundant barcodes representing each of the possible amino acid substitutions in the library. PacBio sequencing was used to link N16 barcode to scFv genotype as previously described^68,69^.

Mutation effects on CGG-binding affinity and scFv surface expression were determined by FACS and sequencing as previously described^68,69^. Briefly, yeast libraries were induced for surface expression, and labeled with a FITC-conjugated anti c-Myc antibody (Immunology Consultants Lab, CYMC-45F) to label for scFv surface expression, or anti-c-Myc antibody and biotinylated CGG (Exalpha IgY-B) followed by PE-conjugated streptavidin (Thermo Fisher S866) to label for CGG-binding. CGG incubations were set up across 10-fold ligand concentrations from 10^−6^ to 10^−13^ M, plus a 0 M baseline sample. Library cells were sorted into four bins of Myc-FITC (for expression) or SA-PE (for CGG binding) as described^68,69^, plasmid extracted from outgrown cells from each sort bin, and barcode counts in each FACS bin were determined via 50 bp single end sequencing on an Illumina NextSeq. Illumina sequencing counts were processed into mutant effects on surface expression and CGG-binding affinity as detailed in https://github.com/jbloomlab/Ab-CGGnaive_DMS/blob/main/Titeseq-modeling.ipynb.

#### Negative-stain electron microscopy (EM)

IgY, either alone or in complex with clone 2.1 Fab, was diluted to ~0.02 mg/ml in Tris-buffered saline and applied to plasma-cleaned, carbon-coated 400 mesh grids. Grids were stained with 2% (w/v) uranyl formate for 30-60 s and blotted. Imaging was performed on an FEI Tecnai Spirit operating at 120-keV, and micrographs recorded with an FEI Eagle 4k CCD camera. Data were processed using Relion 3.0^70^ or cryoSPARC v3.2^71^.

#### Cryo-EM

IgY was incubated with a threefold molar excess of clone 2.1 Fab at room temperature for 5 hours. The final concentration of the complex was diluted to 0.06 – 1.0 mg/ml for vitrification. To aid with sample dispersal on the grid, the complex was mixed with lauryl maltose neopentyl glycol (final concentration of 0.005 mM; Anatrace) and deposited on plasma-cleaned Quantifoil 1.2/1.3 300 mesh grids. A Thermo Fisher Vitrobot Mark IV set to 4°C, 100% humidity, 10 s wait time, and a 3-s blot time was used for the sample vitrification process.

Data were collected using Leginon^72^ over 3 separate imaging sessions on a Thermo Fisher Talos Arctica operating at 200 keV and equipped with a Gatan K2 Summit direct electron detector. Combined movies were aligned and dose weighted using MotionCor2^72^. Aligned frames were imported into cryoSPARC v3.2 and the contrast transfer function (CTF) was estimated using GCTF^73^. Particle picking was done by automated picking using templates created from an initial round of 2D classification, then extracted and subjected to multiple rounds of 2D classification for cleaning. An ab initio volume was generated, followed by 3D classification and the best classes were further refined. To further improve the resolution, the maps were subjected to global and local CTF refinements. A mask was created using UCSF Chimera^74^ and cryoSPARC Volume Tools to cover both clone 2.1 Fabs and the central C_H_2 portion of IgY, and used during local refinement without symmetry. A summary of data collection and processing statistics can be found in Supplemental Table 1.

Initial models were generated by fitting coordinates from sAbPred^75^ for the Fab and ColabFold^76^ for the C_H_2 dimer into the cryo-EM map. Several rounds of iterative manual and automated model building and relaxed refinement were performed using Coot 0.9.4^77^, Rosetta Relax^78^ and Phenix real_space_refine^79^. Models were validated using EMRinger^80^ and MolProbity^81^ as part of Phenix software suite. IMGT numbering was applied to the antibody Fab variable light and heavy chains. Final refinement statistics and PDB/EMDB deposition codes can be found in Supplemental Table 1. Buried surface area was calculated as the mean for the two monomers in the asymmetrical structure, calculated using PISA interface list (https://www.ebi.ac.uk/pdbe/pisa/).

#### Western blotting

Truncated IgY-GFP fusion constructs were generated by cloning each Ig domain of the IgY heavy chain into the XhoI and EcoRI sites of pEGFP-C1-PRKAA1 (Addgene plasmid #30305). 2 μg of each construct was transfected into HEK293T cells using standard calcium phosphate transfection protocols. 24 hours, cells were harvested and 20 μg of protein extracts were loaded onto SDS-PAGE gels and transferred to nitrocellulose membranes using standard wet transfer protocols. All membrane incubations were performed on an orbital shaker. 5% BSA in Tris buffered saline-Tween 20 (TBST) was used to block the membrane for 1 h at 4°C. Both 0.5 μg/ml of recombinant clone 2.1 (A40G) and a 1:5000 dilution of anti-GFP antibody (Biolegend, cat# 902605) were diluted in TBST with 5% BSA for primary staining overnight at 4°C on an orbital shaker. A 1:2,000 dilution of HRP anti-human IgG or a 1:10,000 dilution of HRP anti-mouse IgG antibodies were diluted in TBST with 5% milk for secondary staining against clone 2.1 and anti-GFP antibody, respectively. After 1 h incubation at room temperature on an orbital shaker, membranes were washed, ECL substrate (Pierce) was added, and membranes were exposed for 300 s for signal detection on an Azure c300 imaging system (Azure Biosystems).

### Computational and Quantitative Methods

Most plots were generated in Jupyter notebooks, and an index of which plots appear in what notebooks can be found in https://matsen.group/gcreplay/key-files/#manuscript-figures. This pipeline hosted at https://github.com/matsengrp/gcreplay/ and these notebooks form a reproducible artifact describing the analysis.

#### Sequencing data pipeline

BCR sequencing data were processed using a custom Nextflow v24.04.3.5916 pipeline to reconstruct clonal relationships within germinal centers. The pipeline takes, as input, raw MiSeq paired-end sequencing reads, which first trimmed to remove the first three bases using fastx_trimmer^82^, and subsequently combined using pandaseq^83^. The resulting sequences were demultiplexed based on plate and well barcodes using the fastx_toolkit^69^, producing individual files for each 96-well plate. Heavy and light chain sequences were then separated by identifying conserved motifs using cutadapt^84^ with a 20% error allowance. To reduce noise and focus on biologically relevant sequences, each well’s heavy and light chain sequences were collapsed to unique sequences, and low-abundance BCRs (fewer than 5 reads by default) were pruned to generate ranked files.

The filtered BCR light and heavy chain sequences were then merged across all wells, maintaining identifiers of their origins, and subsequently annotated using partis^85^, primarily to identify V(D)J gene segments, and somatic mutations compared to the naive sequence. The annotated BCR’s were then processed to generate comprehensive datasets containing paired heavy and light chain information for each germinal center. With this, the workflow then performs downstream phylogenetic inference with GCtree (described next), and all other downstream analysis on the resulting GC trees.

#### Phylogenetic inference and tree-based analysis

We inferred phylogenies on clonal families using gctree v4.3.0. GCtree uses PHYLIP’s dnapars utility to infer a collection of maximum parsimony (MP) phylogenetic trees on observed sequences. GCtree then uses a data structure called a history sDAG to expand the collection of MP trees found by dnapars, by swapping parsimony-optimal substructures between them^24^. Next, GCtree ranks the resulting collection of maximally parsimonious trees. For our analysis we configured the ranking to first minimize the number of mutations reverting to the naïve ancestral state, then optimize a branching process likelihood, then a context-based Poisson likelihood, and finally minimize the number of unique sequences in the trees to arbitrarily break any remaining ties. The branching process likelihood uses observed abundances of genotypes to implement the intuition that sequences observed with higher abundance should have more mutant offspring. Trees which follow this intuition have greater branching process likelihood^22^. The context-based Poisson likelihood measures how inferred mutations in the tree agree with context-dependent mutation rates and targeting probabilities of an S5F model^24,35^. Dnapars infers ancestral states, marking sites for which multiple ancestral states are possible under maximum parsimony. GCtree attempts to enumerate all possible maximally parsimonious ancestral states for ranking. If this is not possible for a topology found by dnapars, a single maximally parsimonious ancestral reconstruction is chosen.

#### GC selection metrics

NDS was calculated by splitting each GC into root clades (branches that stem directly from the unmutated ancestor) then dividing the number of cells in the largest lineage by the total sequenced cells in that GC. REI is calculated as follows: For each node X in a phylogeny, the REI represents the sum of the number of descendants of node X weighted according to their mutational distance from node X using a decay factor τ = 0.5, such that cells at 0, 1, 2, … nucleotide distance from node X are weighted 1, 0.5, 0.25, …; the sum of weighted descendants is then divided by the total number of cells in the GC. A phylogeny in which all cells have the same sequence therefore has an REI of 1.0. This metric relies on the assumption is that GC B cells cease SHM when undergoing rapid clonal burst-type expansion^25,26^. The NDS and REI scores are computed on these trees using utilities that are part of our computational pipeline stored on GitHub (https://github.com/matsengrp/gcreplay/blob/main/analysis/NDS-LB.ipynb).

#### Calculation of intrinsic mutability

BCR libraries were processed utilizing the pRESTO suite tools^86^. We adhered to the Illumina MiSeq 2x250 BCR mRNA pipeline. Initially, low-quality reads were filtered out, followed by primer masking and unique molecular identifier (UMI) quantification to correct sequencing errors and PCR amplification biases. Sequences were paired, and consensus sequences were constructed for R1 and R2 reads. These consensus sequences were further paired and assembled into contiguous sequences. Finally, identical sequences were collapsed and quantified. Sequences represented by at least two reads were used for downstream analysis.

BLAST was used to identify sequences with full-length matches for the regions around the CRISPR/Cas9-induced indels that defined the passenger alleles using a 90% identity threshold. The corresponding reads were then subject to a series of filters: the identifying sequence could only be found once in the read, the read could only have one additional indel, and the read could have at most 9 mutations and at most 9 N positions. As is typical in the field^35,87^ the SHM model was described in terms of a “substitution probability,” namely the probability of the new base conditioned on there being a substitution, and a per-site mutability estimate of having a mutation at each position.

In order to combine information across multiple runs for each of the heavy and light chains, we used a Poisson modeling strategy that allowed for the read depth and the overall mutation load to differ between experiments, as well as the mutation rate to differ between sites (further details are provided in https://github.com/matsengrp/gcreplay/blob/main/passenger/igh_passenger_aggregate.ipynb). The per-site rates are parameterized using softmax, resulting in a collection of rates across the sites that sum to 1. A small number of *Igk* positions that had > 0.001% Ns (*Igk* nt 295, 306, 307, 319, 321), indicative of potential sequencing errors, were excluded and replaced by the value obtained using from the five-mer model^35^.

#### Bayesian phylogenetic analysis

We performed Bayesian phylogenetic analysis using BEAST v1.10.4. Nucleotide sequences were analyzed under an HKY substitution model with a strict molecular clock and a constant coalescent demographic model. Markov chain Monte Carlo (MCMC) sampling was conducted for 25 million iterations, with trees sampled every 10,000 steps. After discarding the first 96% of samples as burn-in, the remaining 100 trees were used for the push of the past analysis. The XML template file for the BEAST analysis can be found at https://github.com/matsengrp/gcreplay/blob/main/data/beast/beast_templates/constantsize_histlog.template.patch.

#### Affinity fitness landscape modelling

The minimal mathematical model for the distribution of affinities over time was developed as follows: we denote the log_10_ affinity change with respect to naive as *x* and specify an evolution equation for the probability density of affinities *p*(*x, t*) at each time *t*. There are two key parameter functions that influence the time evolution of *p*(*x,t*) in our model. First, a function *q*(*x,y*) represents the mutational flux (mutation rate per unit *x* and *y*) from affinity state *x* to affinity state *y*, and can be specified (up to a multiplicative scale) by weighting the distribution of log_10_-affinity effects measured in DMS by the mutabilities of each mutation measured from the passenger mouse experiment. Second, an *affinity fitness landscape f*(*x*) specifies the intrinsic fitness for a cell with affinity *x*, and is an unknown function of key interest, as it can be used to predict GC competition between cells of different affinities. The interpretation of this fitness is competitive, so that the Malthusian growth rate of a cell with affinity *x* at time *t* is given by *$f(x)-\overset{‾}{f}(t)$,* where *$\overset{‾}{f}(t)$* is the mean fitness in the population at time *t*. Because the affinity distribution evolves through time, $\overset{‾}{f}(t)$ is expected to increase with time, so that a cell with a given affinity becomes less competitive as its competitors tend to improve in affinity. Assuming a large population, the deterministic evolution equation is

(1) $$\frac{\partial }{\partial t}p(x,t)=(f(x)-\overset{‾}{f}(t))p(x,t)+\int_{-\infty }^{\infty }(q(y,x)p(y,t)-q(x,y)p(x,t))dy,$$

where the first term models birth and death, and the second term models mutation into and out of affinity state *x*^41^. Because the replay mouse has monoclonal naive cells starting GCs, we have the initial condition $p\left(x,t_{0}\right)=\delta (x)$, i.e. a Dirac mass at initial time *t*_0_. Note that this equation is nonlinear due to the dependence of the mean fitness $\overset{‾}{f}$ on p, via $\overset{‾}{f}(t)=\int_{-\infty }^{\infty }f(x)p(x,t)dx$. Also note that this is an equilibrium population size model, which can be seen by integrating both sides of the evolution equation (1) over *x*, which shows the time derivative of the normalization of *p* vanishes identically.

We now detail how we solve the evolution equation, fitting it to the time-course experiment data at time points 5, 8, 11, 14, 17, 20, and 70 dpi. We implement a custom Crank-Nicholson numerical PDE solver as a differentiable program^88^ and use maximum likelihood estimation (MLE) to infer the parameters of the evolution equation that best fit the time-course data, chiefly the fitness landscape *f(x)* which we represent nonparametrically. The time-course data 𝒟 consists of a set of sampling times and a sample of affinities at each such time, so $𝒟\subset R_{+}\times 2^{R}$. The sample size of a sample (t,x)∈𝒟 is |𝒳|. The log-likelihood of *f*(*x*) given the time-course data is

(2) $$\text{l}(f)=\underset{(t,𝒳)\in 𝒟}{\sum }\underset{\text{x}\in 𝒳}{\sum }\text{logp}(\text{x},\text{t}).$$

We perform MLE by backpropagating through the PDE solver to maximize the log-likelihood, using a minimal spline penalty to encourage smooth solutions. More details are available in https://github.com/matsengrp/gcreplay/blob/main/analysis/affinity-fitness-response.ipynb.

We now derive a master equation approach for the stochastic extinction probability $r_{T}(x,t)$ of a lineage with affinity state *x* at time *t* before sampling time *T*. This lineage undergoes a continuous-state birth-death-mutation process^89^ according to death rate μ (an additional free parameter), birth rate $\lambda (x,t)=\mu +f(x)-\bar{f}(t)$, and mutation flux q(x,y) to affinity state *y*. Whence the forward master equation is

(3) $$\frac{\partial r_{T}(x,t)}{\partial t}=-(\lambda (x,t)+\mu +Q(x))r_{T}(x,t)+\lambda (x,t)r_{T}(x,t{)}^{2}+\mu +\int_{\infty }^{\infty }q(x,y)r_{T}(y,t)dy,$$

where $Q(x)=\int_{\infty }^{\infty }q(x,y)dy$ is the total mutation intensity. We solve the equation backward in time from final condition $r_{T}(x,T)=0$, again using our custom Crank-Nicholson solver.

Equipped with a numerical solution to this master equation, we compute the ancestral affinity distribution (the distribution conditioned to leave descendants at the sample time *T*) by weighting *p*(*x, t*) by the probability of survival to time *T* and renormalizing

(4) $$\tilde{p_{T}}(x,t)=\frac{\left(1-r_{T}(x,t)\right)p(x,t)}{\int_{\infty }^{\infty }\left(1-r_{T}(y,t)\right)p(y,t)}dy.$$

We used this forward master equation approach to compute the probability $r_{T}(x,t)$ of stochastic extinction before sampling time *T* = 15 dpi or *T* = 20 dpi.

#### Other software

Flow cytometry data were analyzed using FlowJo v. 10. Plots and statistical analyses not included in Jupyter notebooks were generated using Graphpad Prism v. 10. Figures were edited for appearance using Adobe Illustrator. Claude and ChatGPT were used as aids in coding and to improve the readability of certain passages (but not to generate text). All LLM outputs were carefully checked.

## Supplementary Material

Supplement 1

Supplement 2

3

## Data Availability

Jupyter notebooks used for computational analyses can be found at https://matsen.group/gcreplay/key-files/#manuscript-figures. The cryo-EM map and atomic coordinates of clone 2.1 Fab in complex with IgY have been deposited to the Electron Microscopy Data Bank (EMDB) and Protein Data Bank (PDB) under accession codes EMD-70353 and 9ODB, respectively.
