# Supplementary material for: Replaying germinal center evolution on a quantified affinity landscape
Source: bioRxiv. 2025 Jun 5:2025.06.02.656870. Preprint. [Version 1] doi: 10.1101/2025.06.02.656870 (PMC12258878; doi:10.1101/2025.06.02.656870)
Supplement: 3 [file NIHPP2025.06.02.656870v1-supplement-3.pdf]

bioRxiv preprint doi: https://doi.org/10.1101/2025.06.02.656870; this version posted June 5, 2025. The copyright holder for this preprint (which was not certified by peer review) is the author/funder, who has granted bioRxiv a license to display the preprint in perpetuity. It is made available under aCC-BY 4.0 International license.

# SUPPLEMENTAL INFORMATION

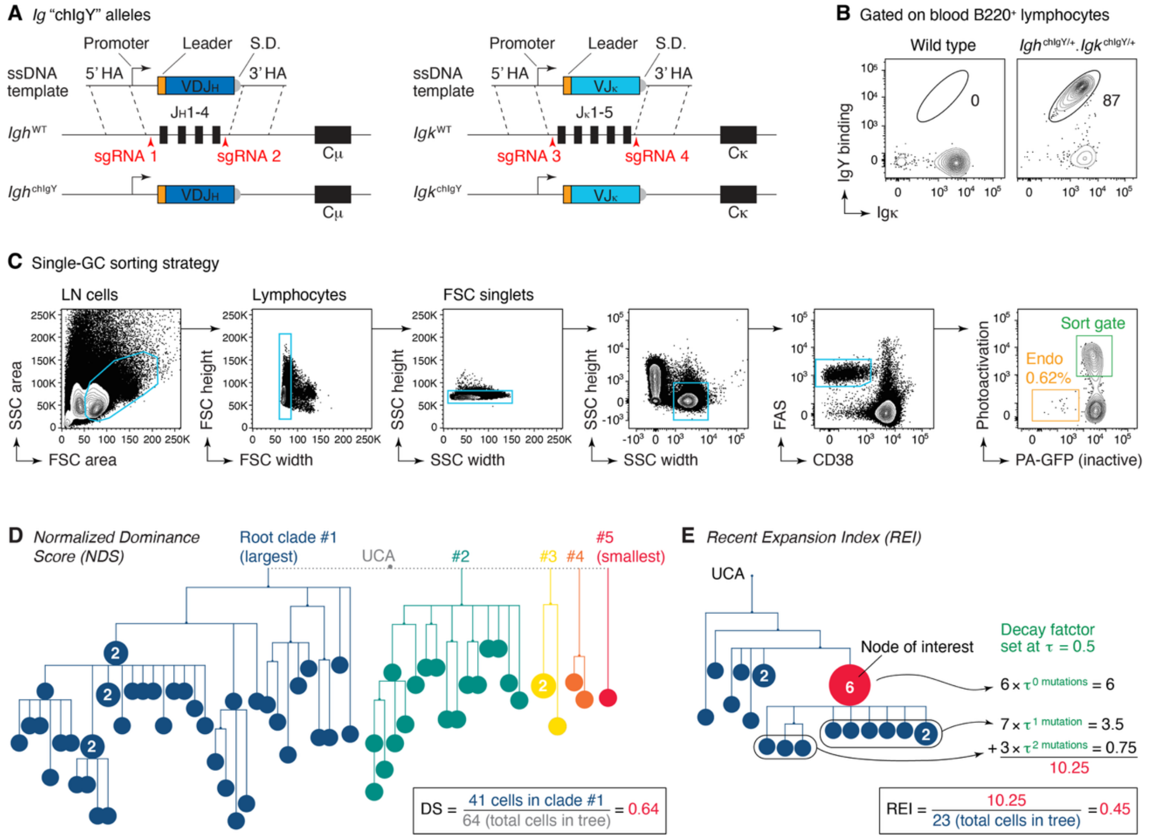


**Figure S1. Parallel replay of germinal center evolution (related to Figure 1).**

**(A)** Design of the *Igh* and *Igk* "chIgY" alleles. CRISPR/Cas9 genome targeting with single-stranded DNA templates was used to replace the endogenous $J_H$ and $J_\kappa$ segments with pre-rearranged V(D)J genes. HA, homology arm; S.D., splice donor.

**(B)** Flow cytometry of blood B cells from wild-type and chIgY mice, showing expression of the rearranged V(D)J genes (inferred from the ability of B cells to bind IgY).

**(C)** Sorting strategy for the parallel replay experiment. FACS plots show a representative LN fragment. Endo, residual endogenous GC B cells derived from the CD23-Cre.*Bcl6*$^{flox/flox}$ host.

**(D)** Schematic representation of the normalized dominance score (NDS) calculation. NDS is equal to the percentage of all cells in a GC that belong to the largest root clade (dark blue). The size of the smaller clades is not included in the calculation.

**(E)** Schematic representation of the recent expansion index (REI) calculation. For each node X in a phylogeny, the REI represents the sum of the number of descendants of node X weighted according to their mutational distance from node X using a decay factor $\tau = 0.5$, such that cells at 0, 1, 2, ... nucleotide distance from node X are weighted 1, 0.5, 0.25, …; the sum of weighted descendants is then divided by the total number of cells in the GC. A phylogeny in which all cells have the same sequence therefore has an REI of 1.0.

bioRxiv preprint doi: https://doi.org/10.1101/2025.06.02.656870; this version posted June 5, 2025. The copyright holder for this preprint (which was not certified by peer review) is the author/funder, who has granted bioRxiv a license to display the preprint in perpetuity. It is made available under aCC-BY 4.0 International license.

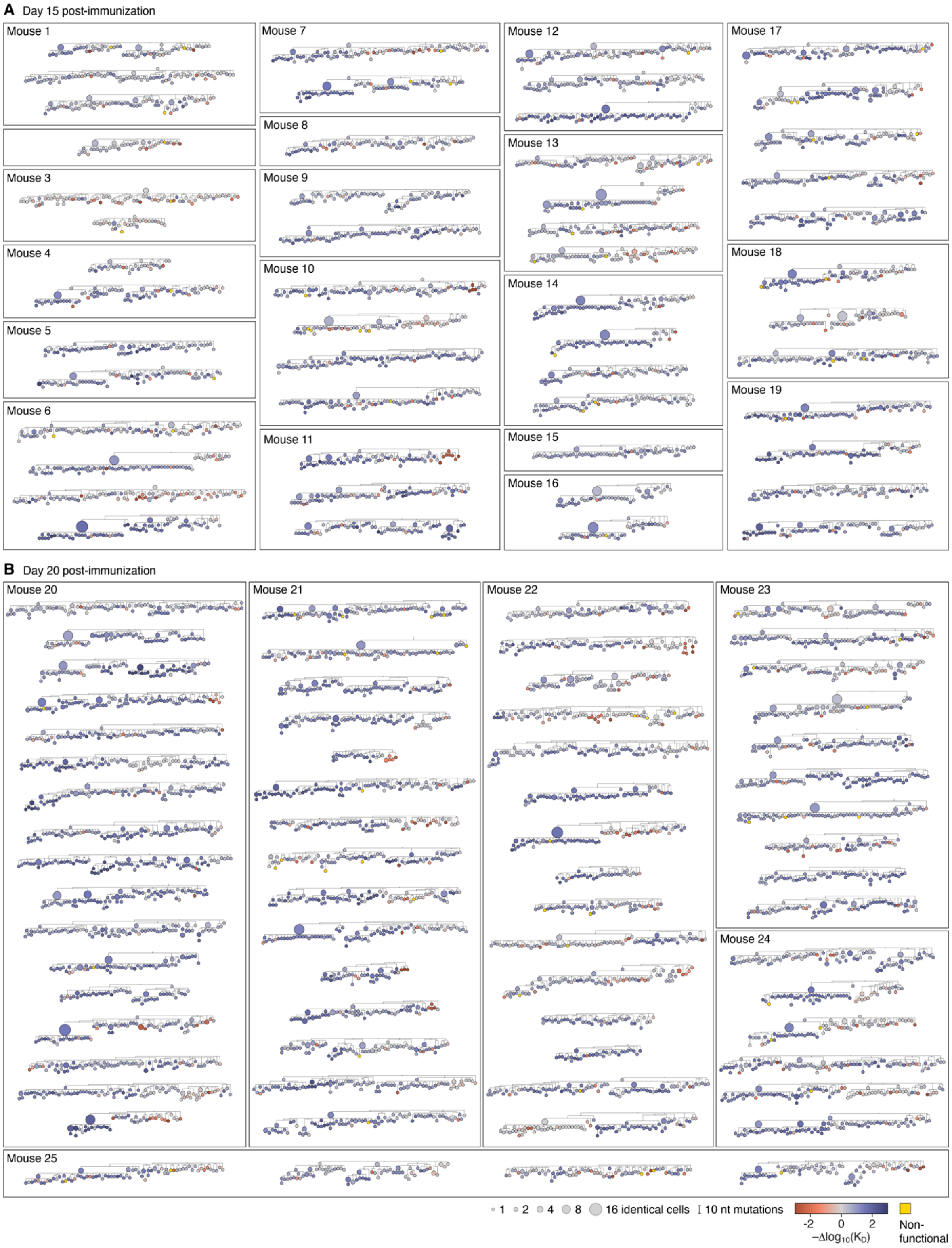


**Figure S2. Individual germinal center phylogenies (related to Figure 1).** Diagrams of all 119 phylogenetic trees inferred from the *Igh+Igk* sequences obtained from each photoactivated GC at **(A)** 15 and **(B)** 20 days post-immunization. Boxes indicate the mouse from which each GC was sorted. Nodes are colored by their relative affinity from the naive precursor (−Δlog10($K_D$)), see Fig. 2 for details.

bioRxiv preprint doi: https://doi.org/10.1101/2025.06.02.656870; this version posted June 5, 2025. The copyright holder for this preprint (which was not certified by peer review) is the author/funder, who has granted bioRxiv a license to display the preprint in perpetuity. It is made available under aCC-BY 4.0 International license.

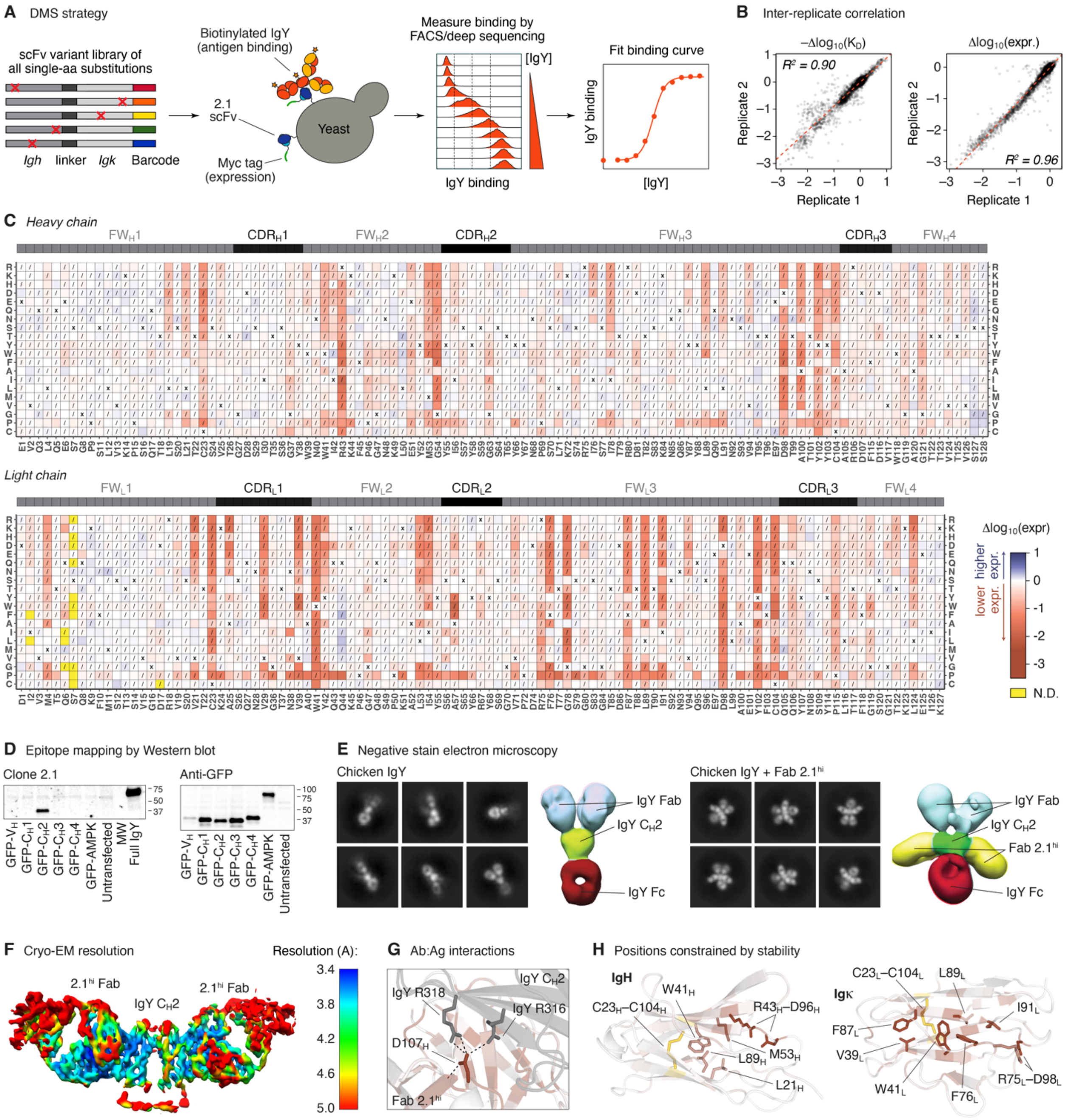


**Figure S3. Deep mutational scanning and structure of clone 2.1 (related to Figure 2).**

**(A)** Experimental setup for the DMS experiment.

**(B)** Correlation in mutation effects on CGG binding affinity (left) and scFv expression level (right) from independently generated and assayed mutant libraries.

**(C)** Heatmap showing the effects of individual amino-acid replacements on surface expression of 2.1 scFv by yeast display. Each square represents a different replacement. Squares with asterisks indicate the original amino acid in clone 2.1. Squares marked with "X" indicate the original amino acid in clone 2.1. Squares with slashes indicate amino acid replacements distant more than one nucleotide mutation from the naïve sequence. Yellow squares were not detected in the DMS experiment. Upper bar shows Kabat CDR and framework (FW) regions in black and gray, respectively. An

bioRxiv preprint doi: https://doi.org/10.1101/2025.06.02.656870; this version posted June 5, 2025. The copyright holder for this preprint (which was not certified by peer review) is the author/funder, who has granted bioRxiv a license to display the preprint in perpetuity. It is made available under aCC-BY 4.0 International license.

interactive version of this heatmap is available at https://matsengrp.github.io/gcreplay/interactive-figures/mutation-heatmaps/naive_reversions_first.html.

**(D)** Coarse mapping of the epitope of clone 2.1. 293T cells were transfected with constructs encoding each Ig domain of $IgY_H$ (or AMPK as a control) fused to GFP. Cell extracts were probed by Western blot with recombinant clone 2.1 mAb (left) or with polyclonal anti-GFP to detect expression of the construct (right). MW, molecular weight ladder, not visible by Western blot.

**(E)** Negative-stain 2D classes and 3D reconstructions (colored by domain/subunit) of unliganded chicken IgY (left) and the $2.1^{hi}$ Fab:IgY complex (right) confirming Fab binding to $C_H2$.

**(F)** Cryo-EM reconstruction of the $2.1^{hi}$ Fab:IgY complex colored by local resolution following IgY $C_H2$ and $2.1^{hi}$Fab local refinement.

**(G)** Key inferred electrostatic interaction between clone 2.1 and IgY. Backbone and side chains colored by mean $\Delta K_D$ for all replacements at each position. Color scale as in Fig. 2A. Potential salt bridge interactions are shown as dotted lines. Mutation of $D107_H$ to anything other than an acidic amino acid ($D107_HE$) results in at least 1 $log_{10}$ decrease in binding affinity.

**(H)** Mapping of selected residues that strongly affect antibody surface expression when mutated. Backbone and side chains colored by mean Δ expression for all replacements at each position. Color scale as in (B); disulfide bridges shown in yellow.

bioRxiv preprint doi: https://doi.org/10.1101/2025.06.02.656870; this version posted June 5, 2025. The copyright holder for this preprint (which was not certified by peer review) is the author/funder, who has granted bioRxiv a license to display the preprint in perpetuity. It is made available under aCC-BY 4.0 International license.

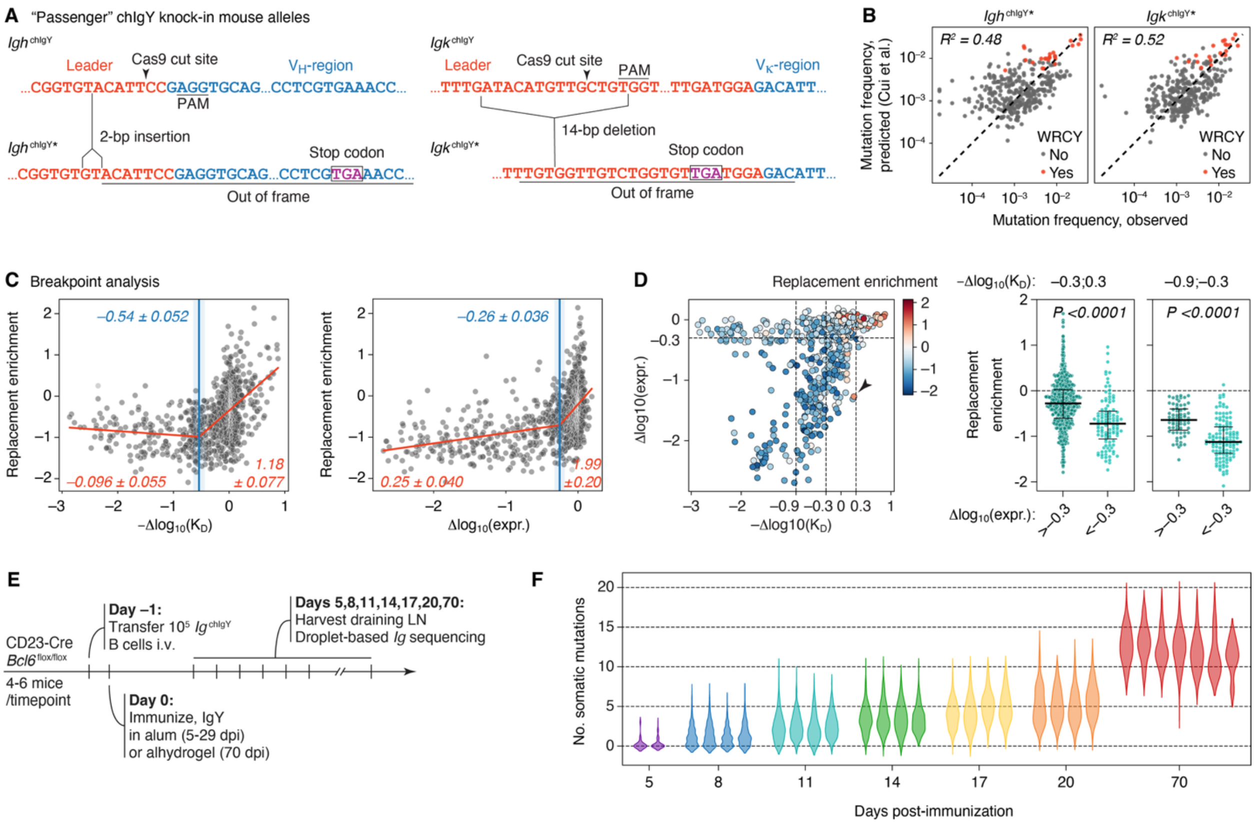


**Figure S4. Selection of individual amino acid replacements (related to Figure 3).**

**(A)** Sequences of the "passenger" *Igh*$^{chIgY\star}$ and *Igk*$^{chIgY\star}$ alleles generated by CRISPR/Cas9-mediated cleavage on the *Ig*$^{chIgY}$ background.

**(B)** Comparison of relative mutation frequencies observed in passenger allele mice *in vivo* with predictions made using the five-mer model[35]. Each symbol represents one nucleotide position of the respective *Ig* sequence. C/G pairs within RGYW AID hotspot motifs are highlighted in red.

**(C)** Segmented regression analysis using the piecewise-regression package in Python. A two-segment fit was chosen by model comparison, and the results of that fit are shown here.

**(D)** Each replacement, plotted in terms of its effect on affinity and expression, and colored according to replacement enrichment. Arrowhead indicates an exceptional replacement that is enriched even though it leads to a marked loss of expression.

**(E)** Layout of the time-course experiment.

**(F)** Distribution of somatic mutations at the indicated time points post-immunization. Each violin represents one mouse. Data for 5-20 dpi were obtained together in a single experiment; data for 70 dpi is from a separate experiment. Two mice (of a total of 9) were excluded from the 70 dpi violin plots for insufficient cell yield; cells from these mice were included in the bulk analysis.

bioRxiv preprint doi: https://doi.org/10.1101/2025.06.02.656870; this version posted June 5, 2025. The copyright holder for this preprint (which was not certified by peer review) is the author/funder, who has granted bioRxiv a license to display the preprint in perpetuity. It is made available under aCC-BY 4.0 International license.

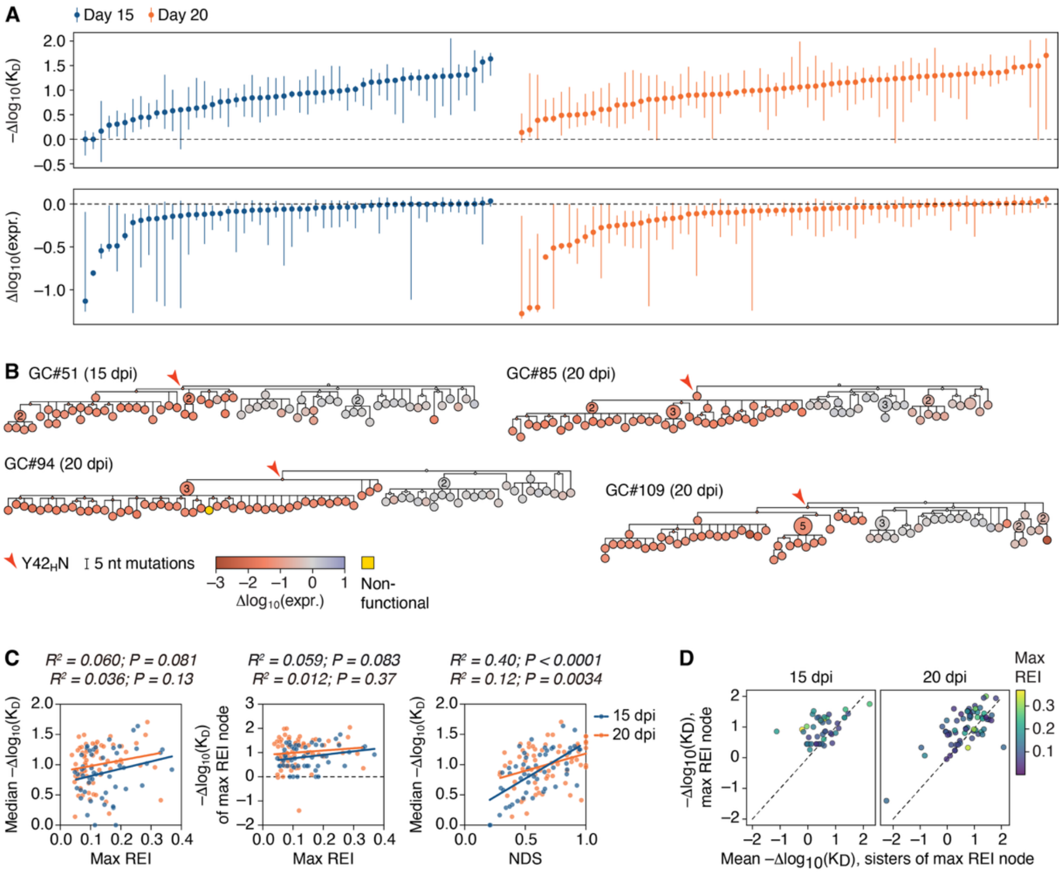


**Figure S5. Drivers of affinity maturation and maintenance of Ig expression (related to Figures 4 and 5).**

**(A)** Median (± range) of Δaffinity (top) and Δexpression (bottom) for all GCs in the replay experiment. Each symbol represents one GC.

**(B)** Examples of GC phylogenies carrying the expression-impairing $Y423_{H}N$ replacement. Trees are colored by Δexpression.

**(C)** Correlations between phylogenetic selection parameters for each GC (max REI and NDS) and median Δaffinity or the Δaffinity corresponding to the sequence of the max REI node). Each symbol represents one GC. Trend lines are for each time point. $R^2$ and P-values are for Pearson correlation and are given for 15 dpi (top row) and 20 dpi (bottom row).

**(D)** Distribution of replay GCs according to the Δaffinity of the max-REI node in the GC and the mean Δaffinity of its "sister" nodes, as defined in Fig. 5G.

bioRxiv preprint doi: https://doi.org/10.1101/2025.06.02.656870; this version posted June 5, 2025. The copyright holder for this preprint (which was not certified by peer review) is the author/funder, who has granted bioRxiv a license to display the preprint in perpetuity. It is made available under aCC-BY 4.0 International license.

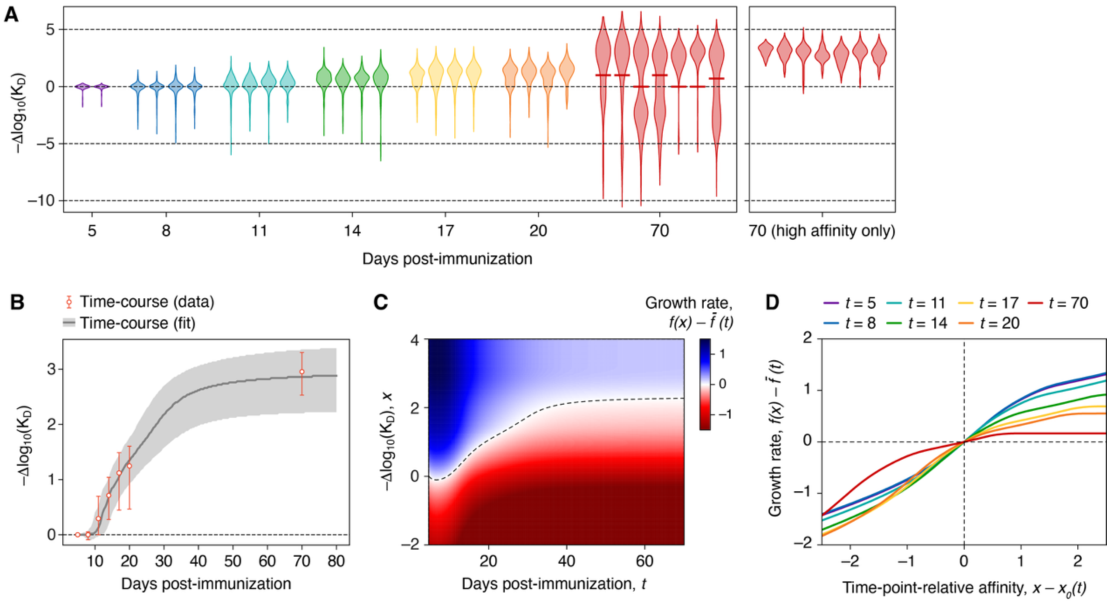


**Figure S6. Progression of affinity over time in bulk-sorted GC B cells (related to Figure 6).**

**(A)** Left**,** distribution of DMS-inferred affinities at the indicated time points post-immunization. Each violin represents one mouse. Data for 5-20 dpi were obtained together in a single experiment; data for 70 dpi is from a separate experiment. Two mice (of a total of 9) were excluded from the 70 dpi violin plots for insufficient cell yield; cells from these mice were included in the bulk analysis. Right, distribution of DMS-inferred affinities at 70 dpi, after exclusion of cells below that persisted in GCs despite having lost all detectable binding to IgY (given that these cells can potentially be explained by co-option of GC lineages to bind other antigens[90,91] in a host mouse strain unable to generate GCs of its own). For each mouse, the upper mode of the distribution was identified, and a threshold was placed manually (red lines in the panel on the right), below which cells were excluded from further analysis.

**(B)** Given the consistency of affinity distributions across individual mice, we aggregated cells from each time point to approximate a single longitudinal sequence of affinity distributions sampled throughout the time-course. Graph shows median and IQR of this distribution overlaid on the prediction generated by the fitness landscape model.

**(C)** The growth rate, given as the intrinsic fitness minus the population mean fitness at each time, is shown in the colormap over time and affinity. The affinity $x_0(t)$ corresponding to population mean fitness at each time is tracked in the dashed line (we call this the time-point-relative affinity).

**(D)** The growth rate, given as the intrinsic fitness minus the population mean fitness, is plotted against the time-point-relative affinity at sampling times. At later times, the fitness response to affinity gains is diminished.

bioRxiv preprint doi: https://doi.org/10.1101/2025.06.02.656870; this version posted June 5, 2025. The copyright holder for this preprint (which was not certified by peer review) is the author/funder, who has granted bioRxiv a license to display the preprint in perpetuity. It is made available under aCC-BY 4.0 International license.

**Supplemental Table 1. Data collection, processing, model refinement and validation**

| **Map** | **IgY + Clone 2.1 Fab (local refinement)** |
|---|---|
| EMDB | EMD-70353 |
| **Data collection** | |
| Microscope | Thermo Fisher Talos Arctica |
| Voltage (kV) | 200 |
| Detector | Gatan K2 Summit |
| Recording mode | Counting |
| Nominal magnification | 36,000x |
| Movie micrograph pixelsize (Å) | 1.15 |
| Dose rate ($e^-$/[(camera pixel)*s]) | 6.95 |
| Number of frames per movie micrograph | 47 |
| Frame exposure time (ms) | 200 |
| Movie micrograph exposure time (s) | 9.5 |
| Total dose ($e^-$/Å$^2$) | 50 |
| Defocus range (μm) | -0.8 to -2.5 |
| **EM data processing** | |
| Number of movie micrographs | 10,007 |
| Number of molecular projection images in map | 346,259 |
| Symmetry | C1 |
| Map resolution (FSC 0.143; Å) | 4.0 |
| Map sharpening B-factor (Å$^2$) | -171 |
| **Structure building and validation** | |
| Number of atoms in deposited model | |
| IgY CH2 | 1,434 |
| Clone 2.1 Fab | 3,400 |
| glycans | 28 |
| MolProbity score | 1.22 |
| Clashscore | 1.36 |
| Map correlation coefficient | 0.73 |
| EMRinger score | 1.63 |
| d FSC model (0.5; Å) | 4.3 |
| RMSD from ideal | |
| Bond length (Å) | 0.007 |
| Bond angles (°) | 1.203 |
| Ramachandran plot | |
| Favored (%) | 94.96 |
| Allowed (%) | 5.04 |
| Outliers (%) | 0.00 |
| Side chain rotamer outliers (%) | 0.00 |
| Cβ outliers (%) | 0.00 |
| PDB | TBD |
